# Polydopamine Nanoparticle‐Mediated Precise Near‐Infrared Optical Stimulation for Cognitive Enhancement

**DOI:** 10.1002/advs.76611

**Published:** 2026-07-28

**Authors:** Yan‐Bo Zhou, Qiong Xue, Su‐Xuan Hou, Ke‐Yao Zhang, Wei‐Tong Pan, Ting‐Ting Zeng, Yu‐Ge Wang, Daqing Ma, Chenguang Zhao, Pan‐Miao Liu, Jian‐Jun Yang

**Affiliations:** ^1^ Department of Anesthesiology Pain and Perioperative Medicine The First Affiliated Hospital of Zhengzhou University Zhengzhou China; ^2^ School of Biomedical Engineering Faculty of Medicine Dalian University of Technology Dalian China; ^3^ Perioperative and Systems Medicine Laboratory, Department of Anesthesiology Children's Hospital Zhejiang University School of Medicine, National Clinical Research Center for Child Health Hangzhou China; ^4^ Chinese Institute for Brain Research Beijing China; ^5^ Division of Anaesthetics, Pain Medicine and Intensive Care Department of Surgery & Cancer Faculty of Medicine Imperial College London, Chelsea and Westminster Hospital London UK; ^6^ Department of Anesthesiology and Perioperative Medicine The First Affiliated Hospital of Nanjing Medical University Nanjing China

**Keywords:** cognitive enhancement, gamma oscillations, neuronal activity, NIR precise stimulation, polydopamine nanoparticles, transcranial neuromodulation

## Abstract

Transcranial neuromodulation faces limitations in precision and depth. This study develops a precise transcranial near‐infrared (NIR) neuromodulation technique using polydopamine nanoparticles (PDA NPs) to address cognitive dysfunction. We synthesize 490 nm PDA NPs as photothermal nanomediators for 1064 nm NIR stimulation and assess their mechanisms and effects in PC12 cells and perioperative neurocognitive dysfunction (PND) model mice. In vitro, PDA NP‐mediated NIR stimulation exhibits a power‐dependent regulatory effect on calcium activity. This activation is mediated through TRPV1, which subsequently enhances mitochondrial membrane potential and intracellular ATP levels. In vivo, this stimulation strategy increases neuronal calcium activity and strengthens gamma oscillations in the hippocampal dentate gyrus (DG). Consequently, the treatment significantly improves spatial memory in both naive and cognitively impaired mice. These findings suggest that PDA NP‐mediated NIR neuromodulation offers a promising, precise therapeutic strategy for treating cognitive dysfunction and related neurological disorders.

## Graphical abstract

Polydopamine nanoparticles enable a precise, non‐genetic, and transcranial neuromodulation strategy via near‐infrared photothermal stimulation. By activating TRPV1 channels, this approach specifically enhances hippocampal gamma oscillations, thereby rescuing spatial memory deficits in models of perioperative neurocognitive disorder. This strategy offers a promising, minimally invasive modality for deep brain stimulation and cognitive recovery.

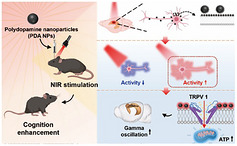

## 1 Introduction

Cognitive dysfunction is a prevalent complication associated with various neurological disorders, such as Alzheimer's disease, Parkinson's disease, and autism, among others [1]. The incidence of cognitive dysfunction has been rising alongside the global aging population, with elderly individuals in the perioperative period particularly vulnerable to perioperative neurocognitive disorders (PND) following surgery [2]. It significantly prolongs the length of hospital stays and results in poor long‐term prognosis [3]. Due to the complex pathogenesis, available treatments are limited, indicating an urgent need for effective therapeutics. In recent years, non‐pharmacological therapies such as electrical, magnetic, and ultrasonic stimulation have emerged as potential treatments for neurological disorders ‐ related cognitive decline. Electrical stimulation, particularly transcranial direct current stimulation (tDCS), has been shown to reduce the incidence of postoperative delirium (POD) in elderly patients undergoing lower limb arthroplasty [4]. Furthermore, both high‐frequency and low‐frequency repetitive transcranial magnetic stimulation (rTMS) were reported to significantly improve memory functions in patients [5, 6], although complications such as headaches and seizures also occurred following these therapies [7]. Therefore, it is imperative to develop safe and effective physical treatment strategies for cognitive‐related neurological disorders, particularly for elderly patients.

Near‐infrared (NIR) light driven transcranial photobiomodulation (PBM) has been shown to improve cognitive function in humans and animals with high safety due to effective photobiological regulation [8, 9]. However, transcranial PBM faces critical limitations of less precision and depth penetration [10]. PBM implemented via invasive cranial approaches also suffers from the issue of photon scattering. In contrast, classical optogenetics—despite its cell‐type specificity—relies on viral transfection and implanted optical fibers, rendering this approach highly invasive and currently restricted to research applications [11]. To overcome these barriers, some injectable light‐sensitive materials have been engineered to deliver NIR targeting the desired region. Notably, a series of up‐conversion nanoparticles (UCNPs) have been developed to convert NIR to visible wavelengths to activate the opsins and modulate neuronal activity with minimal damage [12, 13]. This precise neuromodulation showed improvement in learning and memory in mouse model of PND [14]. However, upconversion‐mediated optogenetics still requires the injection of opsin‐encoding viral vectors to transfect specific neurons for precise neural regulation, and the use of viruses introduces potential biological risks during treatment. Moreover, UCNPs are often doped with rare‐earth elements, and the localized high concentrations of these elements can potentially cause biological toxicity. Therefore, appropriate NIR‐mediator alternatives need to be developed for precise NIR neuromodulation on cognitive enhancement.

Photothermal nanomediators are materials capable of converting light energy into thermal energy [15, 16]. Photothermal nanomediators that convert NIR energy into heat have been proposed to modulate the biological activities of cells [17]. The generated photothermal effect can activate thermosensitive ion channels and effectively regulate neuronal activity in deep brain regions, thereby controlling behavior in mice [18, 19]. This approach leverages the deep tissue penetration of NIR light, while locally administered photothermal agents allow for subregional specificity without the need for permanent hardware implantation. Importantly, this technique overcomes the “scatter‐depth trade‐off” that has long hindered the effectiveness of transcranial photobiomodulation.

Additionally, considering the clinical translation potential of biocompatible photothermal nanomediators, we selected polydopamine nanoparticles (PDA NPs), a melanin‐like material, as the NIR mediator. Inspired by mussel adhesive proteins, dopamine undergoes self‐polymerization under mild alkaline conditions to form PDA NPs. Owing to the conjugated aromatic structure as well as phenolic hydroxyl groups, PDA NPs not only exhibit excellent broadband NIR absorption but also stand out for its high biological safety and strong adhesion properties, making them widely used in the field of biomedicine [20, 21, 22, 23, 24]. Furthermore, PDA NPs have demonstrated efficient neuroprotective capabilities and successful treatment in mice with inflammatory depression by reducing reactive oxygen species (ROS) levels, reversing microglial activation and synaptic damage [25, 26]. Thus, PDA NPs may serve as suitable photothermal nanomediators, enabling precise NIR stimulation with high biosafety in the brain. As hippocampal dysfunction plays a crucial role in cognitive impairment [27], and our previous study indeed showed that strengthening hippocampal neural activity alleviated cognitive impairment in mice [8]. Therefore, the precise modulation of hippocampal neurons is expected to be a promising direction for treating cognitive impairment.

Collectively, these studies suggest that PDA NP ‐mediated precise NIR stimulation can be a promising strategy for the prevention and treatment of cognitive dysfunction‐related diseases. We, therefore, carried out a series of studies and found that 490 nm‐sized PDA NPs were developed as photothermal nanomediators for 1064 nm NIR neuromodulation, demonstrating excellent biosafety in mice. The neuromodulatory effect of PDA NP‐mediated NIR stimulation was investigated in PC12 cells, demonstrating power‐dependent regulation of  cellular excitability. Notably, neuronal activity was modulated via TRPV1. Furthermore, this neuromodulation strategy strengthened the gamma oscillations and then improved the cognitive function in both naive and PND model mice.

## 2 Results

### 2.1 PDA NPs Characterization and Assessment of Biocompatibility

**FIGURE 1:**
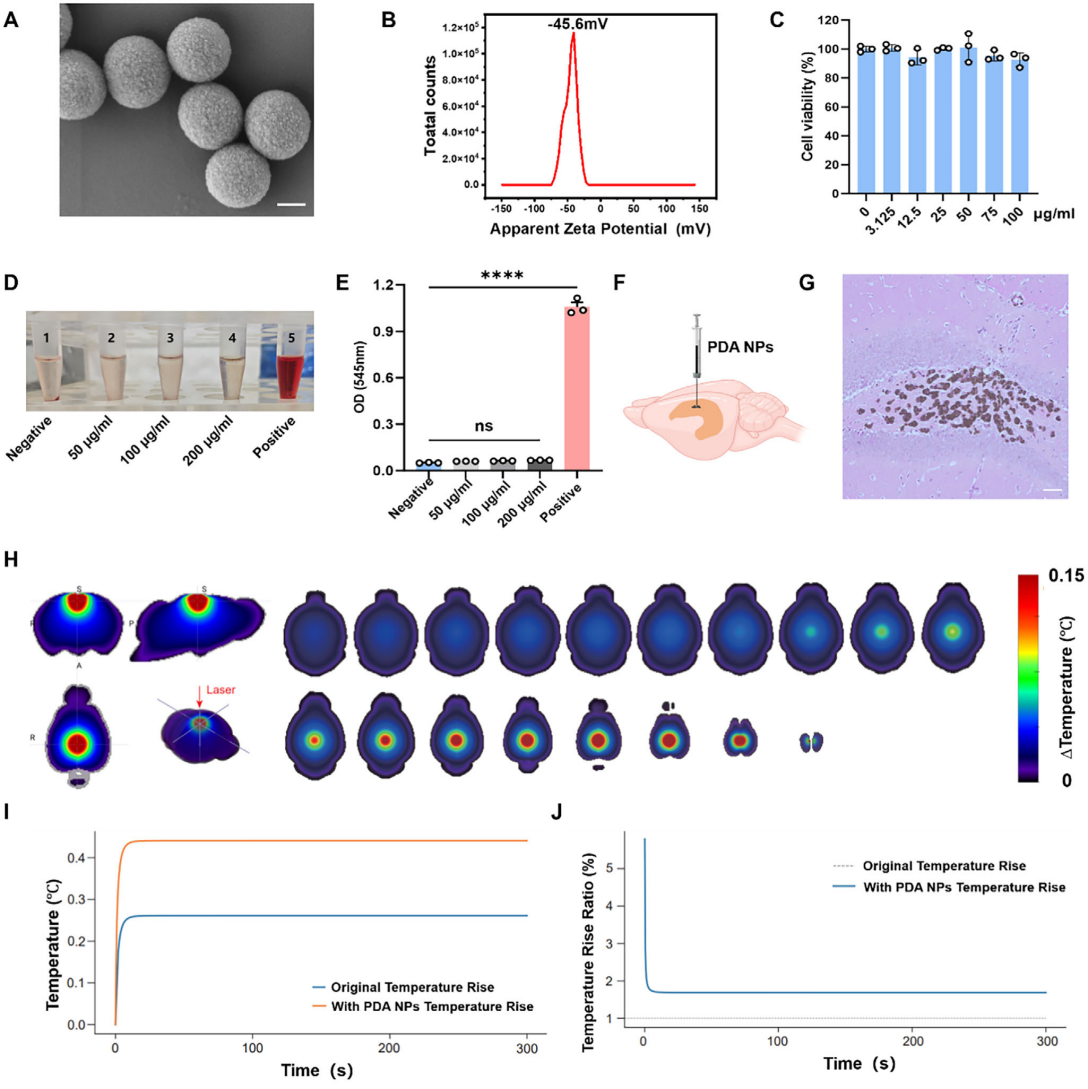
PDA NPs characterization and assessment of biocompatibility. (A) Scanning electron microscope (SEM) images of PDA NPs. Scale bar, 200 nm. (B) Zeta potential analysis of PDA NPs. (C) Cell proliferation and cytotoxicity were assessed by CCK‐8 assay. (F_(6, 14)_ = 1.534, *p* = 0.2380), values are shown as mean ± SD (n = 3/group). (D) Representative picture showing hemolytic reaction of red blood cells in different solutions: PBS (negative), 50 µg/mL PDA NPs, 100 µg/mL PDA NPs, 200 µg/mLPDA NPs, ultrapure water (positive). (E) Bar graphs with dots showing blood compatibility of PDA NPs. (F_(4, 10)_ = 1178, *p* < 0.0001), values are shown as mean ± SEM (n=3 mice/group). (F) Schematic of stereotactic injection of PDA NPs into the hippocampal region. (G) Representative histologic images of DG brain regions after PDA NPs injection. Scale bar, 200 µm. (H) Simulated 3D temperature rise distributions in the mouse brain with PDA NPs injection, showing laser‐induced heating under transcranial illumination. The PDA NPs injection region was modeled as a spherical region with a diameter of 1 mm centered at a dorsoventral depth of 1.9 mm. (I) Simulated temporal profiles of the maximum voxel‐level temperature rise within the PDA NPs‐injected ROI under transcranial 1064 nm NIR irradiation with and without PDA NPs. (J) Relative temperature‐rise ratio calculated from the maximum voxel‐level temperature rise within the PDA NPs‐injected ROI between the PDA NPs‐injected and original conditions. C,E)Results were analyzed by one‐way ANOVA with Dunnett's post‐hoc test, statistically significant differences between negative and positive group are indicated by asterisk: **** *p* <  0.0001.

PDA NPs were prepared by oxidative self‐polymerization of dopamine hydrochloride in an alkaline medium (Figure S1A). As the reaction proceeded, the solution turned from colorless to dark brown. Scanning electron microscope (SEM) images showed that the generated PDA NPs had a monodisperse spherical structure with an average diameter of ∼490 nm (Figure 1A; Figure S1B). The polydispersity index (PDI) of these PDA NPs was 0.043 (Figure S2), and the zeta potential of the PDA NPs was about −46 mV (Figure 1B). In order to further verify the successful formation of PDA NPs, an indole correlation structure located near 1600 cm^−1^ was found with Fourier transform infrared spectroscopy (Figure S3). These results indicated that PDA NPs were successfully prepared.

To assess the biological safety of PDA NPs, PC12 cells were co‐incubated with different concentrations of PDA NPs. After co‐incubating for 24 h, the cytotoxicity was assessed with the cell counting kit‐8 (CCK‐8). Even at a concentration as high as 100 µg/mL, the PDA NPs did not have a significant effect on the proliferation of PC12 cells (Figure 1C). In addition, a hemolytic assay was used to estimate the blood compatibility of PDA NPs. After 4 h of co‐incubation with 4% erythrocytes, PDA NPs with a high concentration of 200 µg/mL showed no detectable hemolysis similar tothat of phosphate buffered saline (PBS) treatment (Figure 1D,E).

Furthermore, the stability tests conducted in various physiological media within 7 days demonstrated that the particle size and PDI of the PDA NPs remained stable, without any significant aggregation, indicating their excellent colloidal stability and anti‐protein adsorption ability (Figure S4). Regarding local retention and diffusion, our results demonstrated that PDA NPs were retained within the target region of the dentate gyrus (DG) for at least three months post‐injection, with partial degradation and minimal diffusion from the injection core to the surrounding tissues (Figure S5).

In addition, to further verify the safety of PDA NPs in vivo, hematoxylin‐eosin (H&E) staining was used to assess the morphology of hippocampal brain region of mice with stereotactic injection of PDA NPs (Figure 1F). PDA NPs caused no significant damage to the surrounding brain tissue and major organs one week after injection (Figure 1G; Figure S6A). To assess neuroinflammation, immunofluorescence analysis of Iba1 and GFAP was performed on hippocampal sections 30 days post‐injection. The results showed that Iba1^+^ cell density in the PDA NPs‐injected group was not statistically different from that of the control group (*p* = 0.063; Figure S7A,B). However, Iba1^+^ cells were observed to encapsulate the PDA NPs, suggesting that these resident immune cells may participate in the gradual clearance of the nanoparticles. Furthermore, no overt GFAP^+^ signals was detected. These findings indicated that PDA NPs did not trigger a sustained neuroinflammatory response one month after administration (Figure S7C,D). Following the injection of PDA NPs into the hippocampal DG, mice exhibited no significant weight loss, and no abnormal pathological changes were observed in the tissue sections of major organs after three months (Figure S6B,C). Overall, PDA NPs exhibited excellent biocompatibility both in vitro and in vivo.

### 2.2 Simulated Temperature Elevation Maps of PDA NPs in the Mouse Brain Under Transcranial NIR Irradiation

Based on the excellent photothermal properties of PDA NPs, we first modeled the temperature rise in mouse brain tissue induced by external NIR irradiation using Monte Carlo simulations. A spherical region with a diameter of 1 mm was embedded within the segmented brain volume as an approximation of the PDA NPs‐injected region. Its center was positioned at a dorsoventral depth of 1.9 mm, matching our in vivo injection depth (DV = −1.9 mm). The corresponding temperature rise (ΔT) in each voxel was calculated by coupling the optical Monte Carlo simulation with the Pennes’ bioheat equation. The total incident optical energy was set to 9.3 J at 1064 nm, corresponding to the cumulative energy delivered during the 300 s irradiation period. The simulation generated a complete 3D temperature map in the absence (Figure S8) and presence (Figure 1H) of PDA NPs, where each voxel was assigned a ΔT value. The following axial temperature maps compared the thermal distributions between the two conditions across successive brain slices. Considering the uncertainty in quantitative optical modeling and the differences between the thermal diffusion model and actual in vivo experimental conditions, the simulation results were primarily presented as relative enhancement ratios to reduce the influence of systematic errors. Comparison of the 3D temperature‐rise maps showed that PDA NPs enhanced the local photothermal response within the simulated injection region. When this region was defined as the ROI, the maximum steady‐state temperature rise within the ROI increased by approximately 0.18°C after PDA NPs injection (Figure 1I), corresponding to a 1.7 fold increase relative to the non‐injection condition (Figure 1J). This maximum voxel‐level temperature rise represents a model‐derived tissue‐scale estimate of the strongest local thermal response formed after PDA NP‐mediated light absorption and subsequent heat conduction and diffusion. These results suggest that PDA NPs can locally enhance and spatially concentrate transcranial NIR‐induced intracranial photothermal effects within and around the injected region.

### 2.3 Photothermal Effect of PDA NPs

**FIGURE 2:**
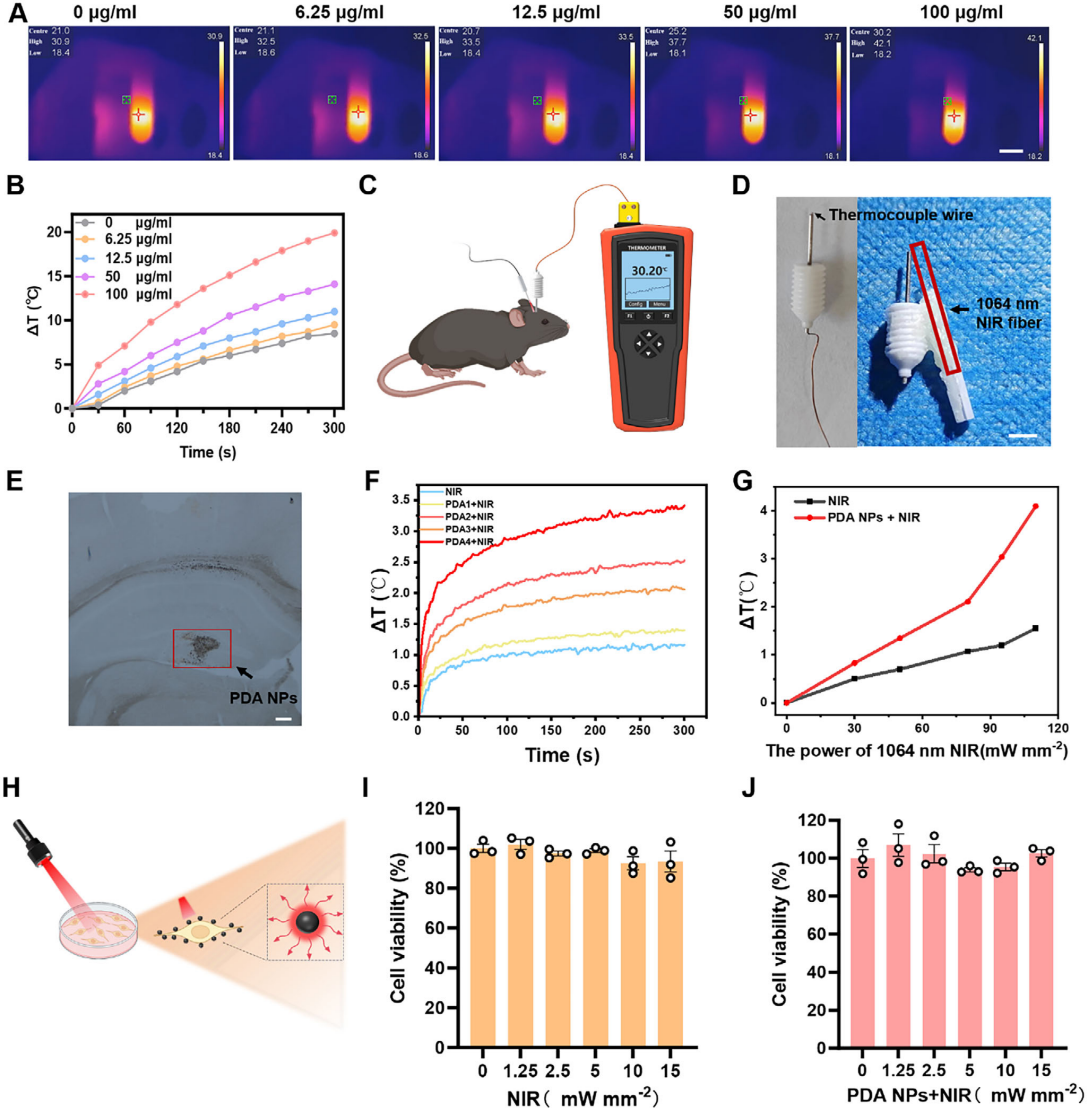
Photothermal properties of PDA NPs. (A) Real‐time infrared thermal imaging of different concentrations of PDA NPs solutions under the NIR irradiation. Scale bar, 10 mm. (B) Temperature change curves of different concentrations of PDA NPs solutions under the irradiation by the 1064 nm laser for 300 s. (C) Schematic of temperature measurements of mice in the DG brain region after PDA NPs injection. (D) Schematic of the recording setup for detecting in vivo temperature elevation induced by 1064 nm NIR light. Scale bar, 3 mm. (E) Representative images of DG brain regions after PDA NPs injection. Scale bar, 200 µm. (F) Temperature change curves in the DG region of mice after injection of different doses of PDA NPs into the DG region at 1064 nm laser irradiation for 300 s. (G) Temperature change curves in the DG region of mice at different power of 1064 nm laser irradiation after 300 s. (H) Schematic representation of PDA NPs after co‐incubation with PC12 cells under 1064 nm NIR irradiation. (I) The cytotoxicity of 1064 nm NIR was assessed by CCK‐8 assay, (F_(5, 12)_ = 1.635, *p* = 0.2247), values are shown as mean ± SEM (n = 3/group). (J) The cytotoxicity of 1064 nm NIR composite PDA NPs was assessed by CCK‐8 assay. (F_(5, 12)_ = 1.609, *p* = 0.2314), values are shown as mean ± SEM (n=3/group). (I, J) Results were analyzed by one‐way ANOVA with Dunnett's post‐hoc test.

On the premise of excellent biocompatibility, we further explored the photothermal properties of PDA NPs with different concentrations under continuous 1064 nm NIR irradiation of 10 mW mm^−2^. As shown in Figure S9, the absorbance of 490 nm PDA NPs at 1064 nm is higher than that of 250 nm PDA NPs, indicating that the absorbance of the larger‐sized PDA NPs at 1064 nm is higher than that of the smaller‐sized particles. By analyzing the heating and cooling curves under 1064 nm NIR irradiation, we calculated the photothermal conversion efficiency (PCE) of 490 nm PDA NPs to be 30.4%, which exceeded that of small‐sized PDA NPs demonstrating an enhanced photothermal conversion ability of large‐sized PDA NPs. During 10 mW mm^−2^ NIR irradiation, the temperature of PDA NPs solutions increased in a concentration‐dependent manner (Figure 2A,B). Specifically, the temperature of the 50 µg/mL PDA NPs solution increased by 2.8°C within 30 s and 14.1°C within 300 s, significantly higher than those of the 0, 6.25 and 12.5 µg/mL groups. Furthermore, the photothermal effect of PDA NPs in vivo was investigated by injecting different doses of PDA NPs into the hippocampal DG region using a thermocouple thermometer (Figure 2C). To record the real‐time intracranial temperature during NIR irradiation, we developed an intracerebral thermometry connector coupled with optical fibers and hollow tubes and for brain implantation (Figure 2D). When fixing the NIR irradiation power at 100 mW mm^−2^ and increasing the injected dose of PDA NPs, the induced temperature rise of the DG region for 300 s was 3.41°C for 50 µg PDA NPs, 2.53°C for 30 µg PDA NPs, 2.06°C for 20 µg PDA NPs, and 1.40°C for 10 µg PDA NPs, all of which were higher than those of the PBS group, which only had a 1.16°C increase (Figure 2E,F). Additionally, when fixing the PDA NPs dose at 50 µg but increasing NIR irradiation power, the induced temperature rise of the DG region for 300 s was 0.83°C for 30 mW mm^−2^, 1.35°C for 50 mW mm^−2^, 2.11°C for 80 mW mm^−2^, and 4.10°C for 110 mW mm^−2^, all of which were higher than those in the PBS group (Figure 2G). Then, the cytotoxicity of NIR irradiation alone as well as PDA NP‐mediated NIR irradiation was detected with CCK‐8 to examine the possible thermal damage (Figure 2H). Results showed that different powers of NIR did not have a significant effect on cell viability (Figure 2I,J). These results indicate that PDA NP‐mediated NIR irradiation has an excellent photothermal effect without thermal damage in vivo and in vitro.

### 2.4 PDA NP‐Mediated NIR Stimulation Enables Power‐Dependent Regulation of Calcium Signaling in PC12 Cells

**FIGURE 3:**
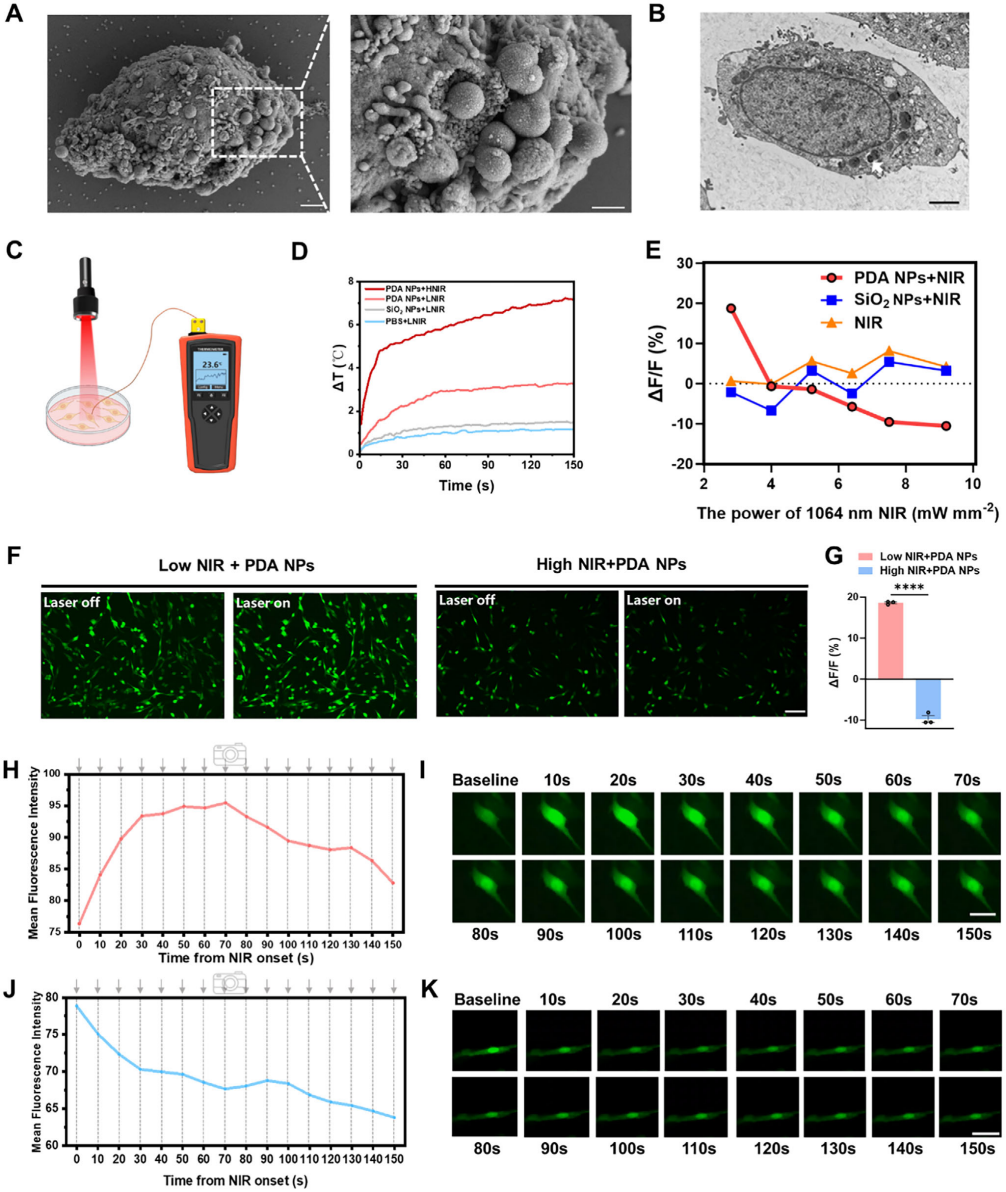
The power‐dependent regulation function of PDA NP‐mediated NIR stimulation on PC12 Cells. (A) Scanning electron micrograph of PDA NPs after 24 h of co‐incubation with PC12 cells. Scale bar, 1 µm, 500 nm. (B) Transmission electron microscopy of PDA NPs with big particle size after 24 h of co‐incubation with PC12 cells. Scale bar, 2 µm. (C) Schematic diagram of temperature measurement when PC12 cells were incubated with PDA NPs and then irradiated using 1064 nm NIR. (D) Change curves of PC12 cells temperature in each group. (E) Change curves of fluorescence intensity for co‐incubation with different nanoparticles at different irradiation powers. (F) Representative images of fluorescence changes in calcium imaging of PC12 cells co‐cultured with PDA NPs in the presence of low‐power NIR (2.8 mW mm^−2^) and high‐power NIR (9.2 mW mm^−2^). Scale bar,100 µm. (G) Intensity of fluorescence changes in calcium imaging of PC12 cells co‐cultured with PDA NPs in the presence of low power NIR (2.8 mW mm^−2^) and high power NIR (9.2 mW mm^−2^). (t_(4)_ = 34.35, *p* < 0.0001), values are shown as mean ± SEM (n=3/group). (H) Representative curve of fluorescence intensity of PC12 cells with time under low‐power NIR stimulation. (I) Representative images of calcium imaging of PC12 cells over time under low‐power NIR stimulation. Scale bar, 25 µm. (J) Representative curve of fluorescence intensity of PC12 cells with time under high‐power NIR stimulation. (K) Representative images of calcium imaging of PC12 cells over time under high‐power NIR stimulation. Scale bar, 50 µm. G) Results were analyzed by unpaired student's *t*‐test, statistically significant differences between low NIR +PDA NPs and high NIR +PDA NPs group are indicated by asterisk: **** *p* < 0.0001.

To explore the action on cellular activity by the thermal effects generated from PDA NP‐mediated NIR stimulation, we further used Fluo‐4AM to label intracellular calcium ion in PC12 cells. After co‐incubation for 24 h, PDA NPs adhered tightly to the surface of PC12 cells, which might be due to the excellent negative charge property of PDA NPs (Figure 3A). Besides, transmission electron microscopy (TEM) image showed that the co‐incubated PDA NPs with 490 nm diameter could hardly enter the interior of PC12 cells, while the relatively smaller PDA NPs with 150 nm diameter were more easily accessible to the interior of cells (Figure 3B; Figure S10). These results demonstrate that 490 nm is a suitable size for PDA NP ‐mediated NIR neuromodulation, as thermosensitive channels are primarily localized on the cell surface. Before stimulating the cells, we measured the temperature of PC12 cells under NIR stimulation with different doses (Figure 3C). Results showed that low‐power NIR stimulation (2.8 mW mm^−2^) increased the temperature of PDA NPs co‐incubated cells by 3.27°C after 150 s, while high‐power NIR stimulation (9.2 mW mm^−2^) increased by 7.16°C. In contrast, NIR stimulation alone or with silica nanoparticles (SiO_2_ NPs) only increased by 1.0–1.5°C after 150 s (Figure 3D). Notably, the calcium fluorescence intensity under 2.8 mW mm^−2^ NIR stimulation in the presence of PDA NPs was able to increase by 18.8% from the basal value in PC12 cells (Figure 3E–I). However, as NIR power was increased to 9.2 mW mm^−2^, the calcium fluorescence intensity decreased by 10.5% from the basal value (Figure 3E,F,G,J,K), indicating that PC12 cells were activated under low‐power NIR and inhibited under high‐power NIR. In contrast, the control group receiving 1064 nm NIRstimulation alone did not show a significant calcium fluorescence intensity change (Figure 3E). This suggests that direct NIR stimulation on PC12 cells is inefficient in heat transfer and thus requires a higher intensity photon volume to increase heat, which may induce tissue damage. Further, to rule out the nano effects of PDA NPs, we synthesized Sio_2_ NPs with a similar particle size to the PDA NPs used in the experiments and conducted the above experiments (Figure S11), and found that SiO_2_ mediated the same power of NIR did not significantly change the intensity of intracellular calcium fluorescence (Figure 3E), and these results suggest that PDA NP‐mediated NIR stimulation modulates activity of PC12 cells depending on a photothermal effect. To investigate the potential chemical decomposition of PDA NPs under NIR irradiation, we measured the release of dopamine (DA) monomer in the supernatant. As shown in Figure S12, the DA concentration in the control group was 0.196 µM, while the concentration in the low‐power NIR group remained at baseline levels (0.166 ± 0.007 µM). Although high‐power NIR irradiation led to a marginal increase in DA concentration (0.257 ± 0.016 µM), this specific intensity was not employed in our in vivo biological experiments. These results demonstrate that PDA NPs maintain high chemical stability and do not release significant amounts of dopamine under our experimental conditions.

### 2.5 PDA NP‐Mediated NIR Stimulation Modulates Mitochondrial Energy Metabolism

**FIGURE 4:**
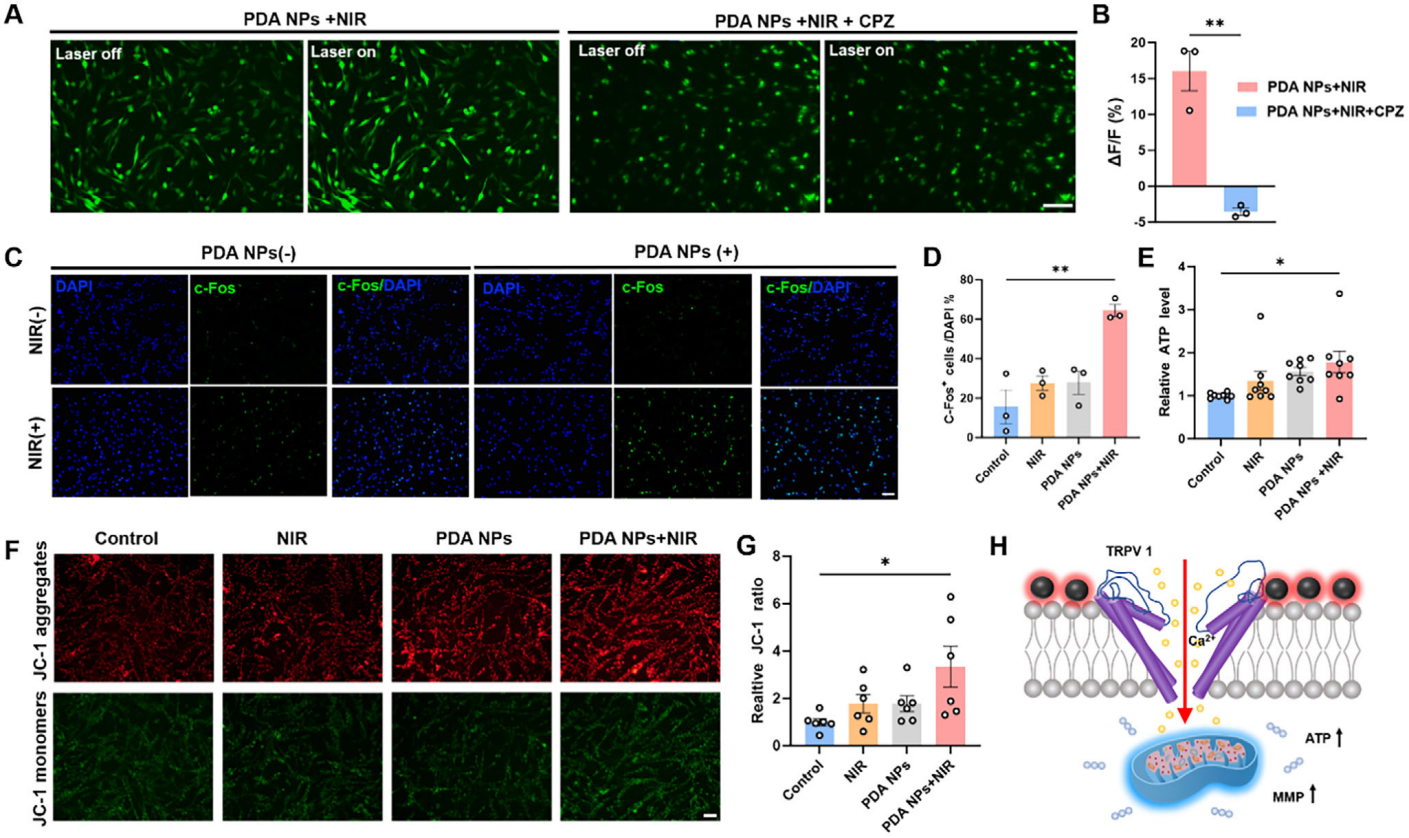
PDA NP‐mediated NIR stimuation modulates mitochondrial energy metabolism. (A) Calcium imaging of PC12 cells co‐cultured with PDA NPs under low‐power NIR and Capsazepine. Scale bar,100 µm. (B) Intensity of fluorescence changes in calcium imaging of PC12 cells co‐cultured with PDA NPs in the presence of low‐power NIR and capsazepine. (t_(4)_ = 7.023, *p* = 0.0022), values are shown as mean ± SEM (n = 3/group). (C) Representative immunofluorescence of c‐Fos under different experimental conditions. Scale bar,100 µm. (D) Number of c‐Fos^+^ neurons in different experimental conditions. (F_(3, 8)_ = 13.48, *p* = 0.0017), values are shown as mean ± SEM (n = 3). (E) Level of ATP in PC12 cells under different experimental conditions. (F_(3, 28)_ = 3.642, *p =* 0.0246), values are shown as mean ± SEM (n = 8/group). (F) Representative images of JC‐1 signaling in PC12 cells detected by fluorescence microscopy under different experimental conditions. Scale bar,100 µm. (G) Fluorescence intensity of JC‐1 signal in different group. (F_(3, 20)_ = 3.699, *p* = 0.0288), values are shown as mean ± SEM (n = 6/group). (H) Mechanisms of the effects of PDA NP‐mediated NIR stimulation on cellular mitochondrial production. B) Results were analyzed by unpaired student's *t*‐test. D, E, G) Results were analyzed by one‐way ANOVA with Dunnett's post‐hoc test. Statistically significant differences are indicated by asterisk: ** *p* < 0.01, * *p* < 0.05.

TRPV1 has been reported to be a cation channel that mediates Calcium influx through temperature sensing. We verified the expression of TRPV1 on PC12 cells (Figure S13). In order to investigate the role of TRPV1 in the PDA NP‐mediated NIR modulation, we subsequently used the TRPV1 antagonist capsazepine (CPZ) in the group of PDA NPs mediated low‐power NIR and found that the fluorescence intensity of calcium was significantly decreased (Figure 4A,B), suggesting that PDA NP‐mediated low‐power NIR stimulation might trigger calcium influx through TRPV1. Next, we similarly found that the number of c‐Fos^+^ cells in the PDA NP‐mediated low‐power NIR group was significantly greater than that in the other groups (Figure 4C,D). More importantly, the ATP level was also significantly higher in this group compared to other control groups (Figure 4E), which is consistent with the above results. We next used a fluorescent probe for JC‐1 to detect any changes in mitochondrial membrane potential in PC12 cells after the intervention. We were surprised to find that the mitochondrial membrane potential of PC12 cells in the PDA NP‐mediated low‐power NIR group was significantly elevated compared to other control groups (Figure 4F,G). Above all, the increase of mitochondrial energy metabolism may go through the following mechanism: PDA NPs mediate the generation of photothermal effect by NIR, thereby activating the TRPV1 and causing calcium ion influx. The flowing calcium ion can enter the interior of the mitochondria, thereby enhancing the function of the mitochondria and the release of ATP (Figure 4H).

### 2.6 PDA NP‐Mediated NIR Stimulation Activates Neurons in Hippocampus

**FIGURE 5:**
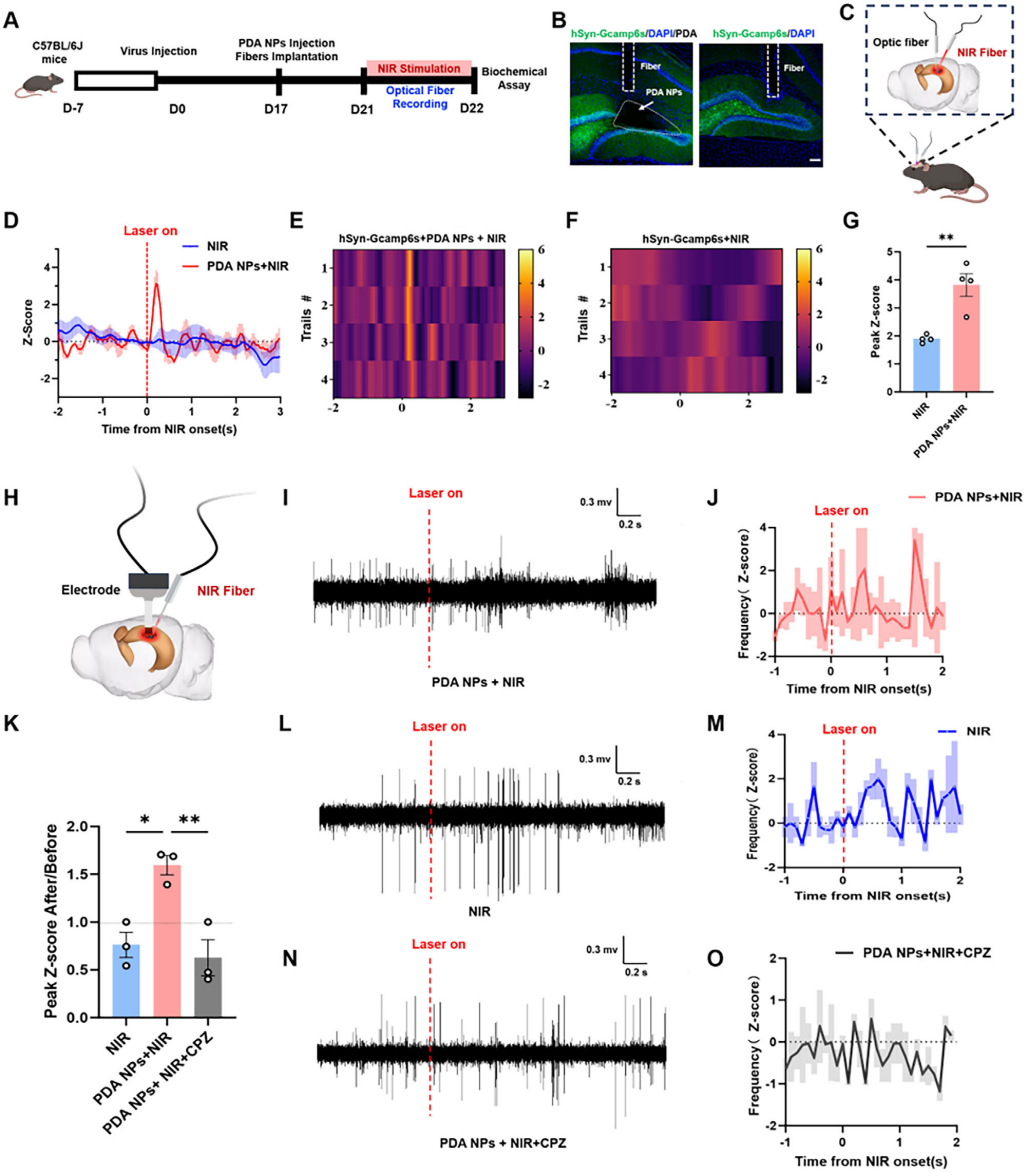
PDA NP‐mediated NIR stimulation activates neurons in hippocampus. (A) Schematic representation of the experience of mice undergoing fiber‐optic recording. (B) Representative images of rAAV‐hSyn GCaMp6s virus and PDA NPs or PBS injected in the DG brain region. (C) Schematic representation of a mouse brain subjected to fiber‐optic recording and irradiated with 1064 nm NIR. (D) Z score showing Calcium ion transients evoked by PDA NP‐mediated 1064 nm NIR stimulation. (E, F) Heatmaps showing Calcium ion transients evoked by 1064 nm NIR. (G) Peak Z‐Score of DG neurons in PDA NP‐mediated NIR group and NIR‐only group. (t_(6)_ = 4.667, *p* = 0.0034), values are shown as mean ± SEM (n = 4 mice/group). (H) Schematic of a mouse brain subjected to in vivo electrophysiological recordings and NIR irradiation at 1064 nm. (I, J) Representative traces and frequency of spikes in DG neurons evoked by PDA NP‐mediated 1064 nm NIR stimulation. (K) The ratio of Peak Z‐score of spike frequency of DG neurons before and after NIR irradiation in the PDA NPs + NIR group, NIR group and PDA NPs + NIR + CPZ group. (F_(2, 6)_ = 13.06, *p* = 0.0065), values are shown as mean ± SEM (n = 3 mice/group). (L, M) Representative traces and frequency of spikes in DG neurons evoked by 1064 nm NIR stimulation. (N, O) Representative traces and frequency of spikes in DG neurons evoked by PDA NPs+1064 nm NIR+CPZ. G) Results were analyzed by unpaired student's *t*‐test. (K) Results were analyzed by one‐way ANOVA with Bonferroni's post‐hoc test. Statistically significant differences are indicated by asterisk: ** *p* < 0.01, *p < 0.05.

Having demonstrated that PDA NP‐mediated low‐power NIR activates PC12 cells in vitro, we further explored it could activate neurons in mice. To avoid scalp overheating, we monitored the temperatures of the mouse cortex and the target hippocampal DG region during PDA NP‐mediated NIR stimulation using a thermocouple thermometer (Figure S14A). Under 100 mW mm^−2^ NIR irradiation, PDA NPs induced a temperature rise of 3.4°C in the DG, while the superficial cortex temperature remained within a safe range (Figure S14B), achieving targeted deep‐brain heating. In contrast, without PDA NPs, achieving equivalent heating in the deep brain would require NIR powers that elevate the superficial cortex temperature by more than 10°C (Figure S14B). This exceeds the safety threshold for neural tissue, underscoring that PDA NPs are indispensable transducers for safe and precise deep‐brain neuromodulation. To affirm whether PDA NP‐mediated NIR stimulation could activate neurons, we performed in vivo calcium signal recording in the hippocampus of mice using rAAV‐hSyn‐GCaMp6s‐WPRE and a self‐made composite optical fiber (Figure 5A; Figure S15). As expected, PDA NPs and fiber were both delivered into the hippocampal DG (Figure 5B). The calcium signals were recorded during NIR stimulation mediated by PDA NPs (Figure 5C). The baseline of calcium signals before NIR turned on for alone NIR group and PDA NP‐mediated NIR group were similar (Figure 5D), suggesting that the injection of PDA NPs had negligible effects on basal neuronal activity. When NIR was turned on, the calcium signals in PDA NP‐mediated NIR group were suddenly increased, but no change in alone NIR group (Figure 5D–G). To further investigate the neuronal firing activity during NIR stimulation, we used a self‐made multi‐channel electrode and fiber complex to record the local neuronal firing during the NIR stimulation mediated by PDA NP (Figure 5H; Figure S16). Compared with the baseline before applying NIR, the peak Z‐score of spikesfrequency in the DG region mediated by PDA NPs increased 1.6 times (Figure 5I–M). These results suggest that PDA NP‐mediated NIR stimulation has the capability to activate neurons in the hippocampal DG region of mice.

### 2.7 PDA NP‐Mediated NIR Stimulation Enhances Hippocampal Gamma Oscillations and Improves Cognitive Function in Naive Mice

**FIGURE 6:**
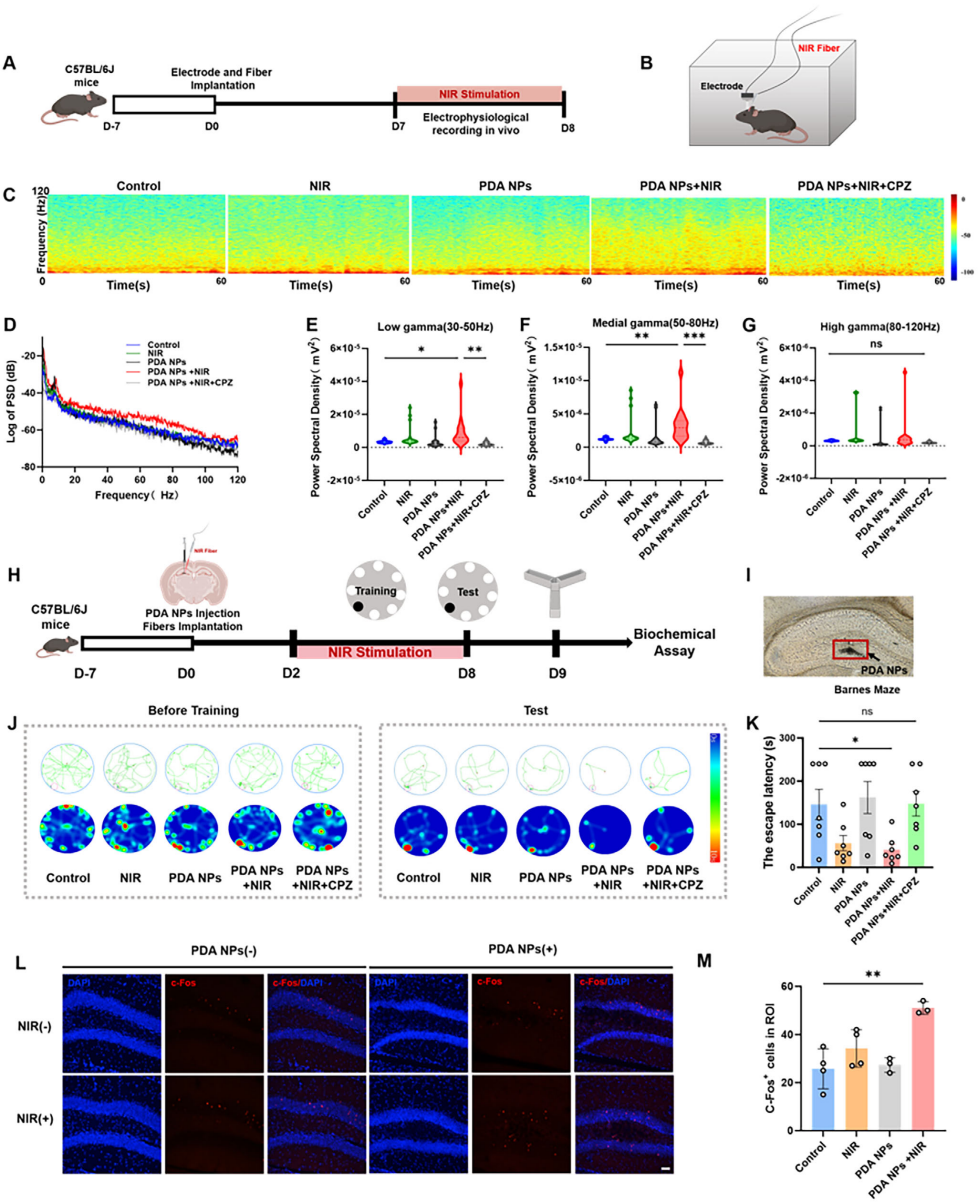
PDA NP‐mediated NIR stimulation enhances hippocampal gamma oscillations and improves cognitive function in naive mice. (A,B) Experimental timeline and schematic diagram of mice making in vivo electrophysiologic recordings. (C) Representative heat‐maps of the power spectogram in the DG. (D) The power spectral density of local field potential in the DG. (E) Quantification of average low gamma, (F_(4, 75)_ = 4.643, *p* = 0.0021) (F) medial gamma (F_(4, 75)_ = 5.588, *p* = 0.0005) and (G) high gamma in the DG in different groups (F_(4, 75)_ = 1.863, *p* = 0.1257), values are shown as mean ± SEM (n = 16 from 4 mice/group). (H) Timeline of PDA NPs injection and irradiation with NIR and behavioral testing. (I) Representative images of PDA NPs injected into the DG region. (J) Representative trajectory plots and heat‐maps for each group at pre‐training and testing of the Barnes maze. (K) The escape latency in the test in each group. (F_(4, 30)_ = 4.206, *p* = 0.008), values are shown as mean ± SEM (n = 7 mice/group). (L) Representative immunofluorescence of C‐Fos under different experimental conditions in DG region. Scale bar,50 µm. (M) Number of c‐Fos^+^ neurons in different experimental conditions. (F _(3, 10)_ = 10.00, *p* = 0.0023), values are shown as mean ± SEM (n = 3‐4 mice/group). E, F, G) Results were analyzed by one‐way ANOVA with Tukey's post‐hoc test. K, M) Results were analyzed by one‐way ANOVA with Dunnett's post‐hoc test. Statistically significant differences are indicated by asterisk: *** *p* < 0.001, **p < 0.01, *p < 0.05.

In this section, we used complex photoelectrode to study the in vivo local field potential (LFP) in the DG of freely moving mice stimulated by PDA NP‐mediated NIR (Figure 6A,B). Compared with control mice, the power of neural oscillations in the DG in the PDA NP‐mediated NIR‐stimulated mice was significantly increased at the irradiance of 100 mW mm^−2^ (Figure 6C,D; Figure S17). Specifically, the power spectral density of the medial‐ and low‐gamma oscillations increased significantly compared to the control mice in the PDA NP‐mediated NIR‐stimulated mice (Figure 6E,F), but the oscillations of the other frequencies showed no significant changes (Figure 6G; Figure S18). Notably, the power of neural oscillations in the PDA NPs‐only and NIR‐only groups showed no significant differences compared with the control group and PDA NP‐mediated NIR stimulation group. These results demonstrate that PDA NP‐mediated NIR stimulation enhances hippocampal low‐ and medial‐gamma oscillations in naive mice.

Hippocampal neuronal network activity is closely related to cognitive function. To affirm this, we utilized Barnes maze and Y maze to test the spatial learning and memory of these naive mice after PDA NP‐mediated NIR stimulation (Figure 6H). We injected PBS or PDA NPs into the hippocampus of mice and then implanted optical fibers (Figure 6I). After the mice rested for one day, we stimulated the mice with NIR during the training in the Barnes maze, and then tested the mice of each group after 6 days of continuous NIR neuromodulation. As the number of training sessions was increased, all groups of mice were able to find the target hole faster than on the first day (Figure S19A). Mice receiving PDA NP‐mediated NIR stimulation spent less time (41.1 ± 12.2 s) to find the target hole, with a significantly shorter time than that in the control mice (146.0 ± 35.0 s) on the test session (Figure 6J, K). Notably, the NIR‐only group also spent less time (56.2 ± 17.7 s) to find the target hole than control mice, which matches the previous 1064 nm NIR stimulation on healthy human studies [9]. However, Y maze test showed no significant difference in the proportion of alternation entering the arms on the second day after training (Figure S19B,C). Then, c‐Fos was stained with immunofluorescent method to indicate the neuronal activation in the hippocampal region of the mice. Results revealed that only PDA NP‐ mediated NIR stimulation group showed significant increase in the number of c‐Fos^+^ neurons in the DG region, compared to the other groups (Figure 6L,M). To quantitatively assess the spatial precision of this technology, we analyzed the relationship between c‐Fos expression and the radial distance from the injection center. The results showed that c‐Fos^+^ neurons were primarily concentrated within a 100 µm‐150 µm radius of the injection center and decayed rapidly as the distance increased (Figure S20). Given the full width at half maximum (FWHM) of the c‐Fos activation profile was approximately 275 µm, the effective stimulation radius was defined as 137.5 µm. This quantitative result is highly consistent with the empirically observed spread of c‐Fos^+^ cells. Furthermore, no significant c‐Fos expression was observed in the control groups. These findings indicate that neural activation is precisely confined to the intersection zone of light and nanoparticles, thereby achieving neuromodulation with sub‐regional precision.

Furthermore, to establish a causal link between TRPV1 activation and cognitive outcomes, we administered CPZ into the DG of mice 30 min prior to NIR stimulation. Compared with the baseline before the stimulation of NIR, local neuronal discharges in the PDA NPs‐NIR‐CPZ group were significantly lower than those in the PDA NP‐mediated NIR stimulation group during irradiation (Figure 5K,N,O). LFP recordings in freely moving mice showed no significant difference in low‐gamma and medial‐gamma oscillations between the PDA NPs‐NIR‐CPZ group and the control group (Figure 6E,F). Additionally, TRPV1 blockade completely neutralized the cognitive improvements associated with the PDA NP‐mediated NIR stimulation group. In the Barnes maze, the escape latency for the PDA NPs‐NIR‐CPZ group (147.4 ± 28.3 s) was comparable to that of the control group (146.0 ± 35.0 s) (Figure 6K). These pharmacological findings demonstrate that even under equivalent photothermal conditions, the neurotherapeutic effects are lost if TRPV1 channels are inactivated, confirming the specificity of this neuromodulatory pathway.

### 2.8 PDA NP‐Mediated NIR Stimulation Improves Cognitive Function of PND Mice

**FIGURE 7:**
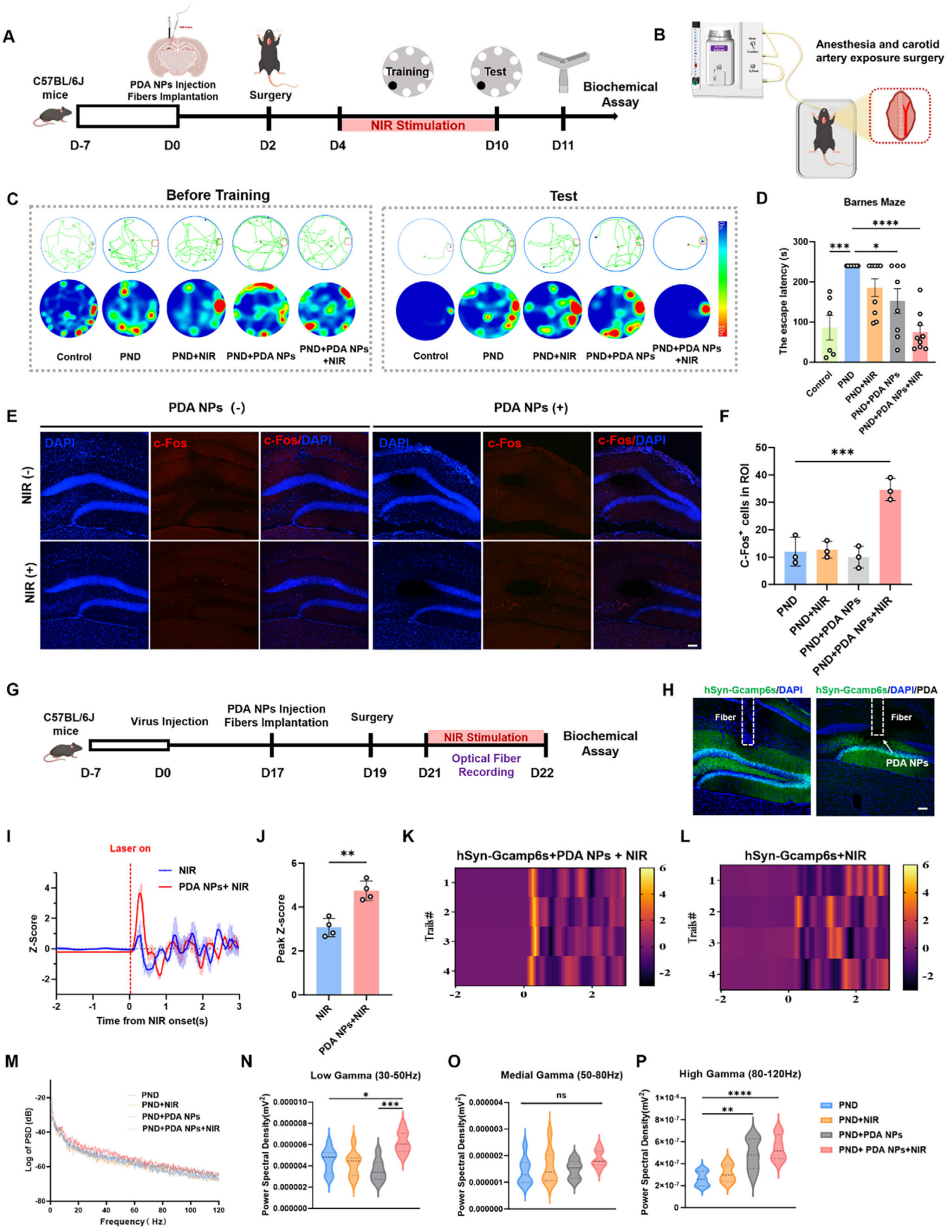
PDA NP‐mediated NIR stimulation improves cognition of PND mice. (A) Timeline of PDA NPs injection, surgery, irradiation with NIR, and behavioral testing. (B) Schematic diagram of the PND procedure. (C) Representative trajectory plots and heat maps for each group at pre‐training and testing of the Barnes maze. (D) The escape latency in the test in each group. (F_(4, 36)_ = 10.49, *p* < 0.0001), values are shown as mean ± SEM, (n = 6‐8 mice/group). (E) Representative immunofluorescence of c‐Fos under different experimental conditions in the DG region. Scale bar, 100 µm. (F) Number of c‐Fos^+^ neurons in different experimental conditions, (F_(3, 8)_ = 23.22, *p* = 0.0003), values are shown as mean ± SEM, (n = 3 mice/group). (G) Timeline of the experience of PND mice undergoing fiber‐optic recording. (H) Representative images of rAA‐hSyn GCaMp6s virus and PDA NPs or PBS injected in the DG region. Scale bar, 100 µm. (I) Z score showing Calcium ion transients evoked by PDA NP‐mediated 1064 nm NIR or NIR‐only. (J) Peak Z‐Score of DG neurons in PDA NP‐mediated NIR stimulation group and NIR‐only group, (t_(6)_ = 5.513, *p* = 0.0015), values are shown as mean ± SEM (n = 4 mice/group). (K) Heat‐maps showing Calcium ion transients evoked by PDA NP‐mediated 1064 nm NIR stimulation. (L) Heat‐maps showing Calcium ion transients evoked by 1064 nm NIR. (M) The power spectra of local field potential in the DG. (N‐P) Quantification of average low gamma (F_(3, 28)_ = 8.628, *p* = 0.0003), medial gamma(F_(3, 28)_ = 1.497, *p* = 0.2368), and high gamma (F_(3, 28)_ = 12.88, *p* < 0.0001)in the DG for each group, values are shown as mean ± SEM (n=8 from 4 mice/group). D, F) Results were analyzed by one‐way ANOVA with Dunnett's post‐hoc test. J) Results were analyzed by unpaired student's *t*‐test. N‐P) Results were analyzed by one‐way ANOVA with Tukey's post‐hoc test. Statistically significant differences are indicated by asterisk: **** *p* < 0.0001, *** *p* < 0.001, **p < 0.01, *p < 0.05.

We next explored whether the photothermal biological effect generated by PDA NP‐mediated NIR stimulation can improve cognitive function in PND mice. Barnes maze and Y maze were applied to test the spatial learning and memory of these PND mice after irradiation. We first injected PDA NPs or PBS into the hippocampus and implanted optical fibers, and then modeled PND mice by carotid artery exposure (Figure 7A,B). After the mice rested for one day, the Barnes maze training and NIR stimulation were conducted as previously described.While all groups showed improved performance over time except for the PND group (Figure S21A), PDA NP‐mediated NIR stimulation group spent less time (74.8 ± 16.4 s) to find the target hole, with a significantly shorter time than that in the PND mice (240s) on the test session (Figure 7C,D). Interestingly, PDA NPs group spent less time (152.6 ± 31.1 s) to find the target hole than PND group, potentially due to the anti‐inflammatory properties of PDA NPs. However, Y maze test showed no significant difference on the proportion of alternation entering the arms on the second day after training (Figure S21B,C). Subsequent immunofluorescence staining of the hippocampal DG region of mice showed that only in PDA NP‐mediated NIR stimulation group, the expression of c‐Fos in the DG region was significantly increased compared with that in the PND group (Figure 7 E, F). We further performed in vivo calcium signal recording in the hippocampus of mice using rAAV‐hSyn‐GCaMp6s‐WPRE and a self‐made composite optical fiber (Figure 7G,H; Figure S15). We found that only in the presence of PDA NPs, NIR power irradiation with 100 mW mm^−2^ significantly increased calcium activity of neurons in the hippocampal DG region of PND mice (Figure 7I–L). The above results indicate that PDA NP‐mediated NIR stimulation activates neurons in the hippocampal DG region and alleviates cognitive deficits in PND mice.

We further analyzed the LFP in the DG region of all groups of mice (Figure S22A,B). Compared with PND group, the power of neural oscillations in the DG in the PDA NP‐mediated NIR stimulation group was significantly increased at the irradiance of 100 mW mm^−2^ (Figure 7M; Figure S22C,D). Among them, the power spectral density of the high‐ and low‐gamma oscillations increased significantly compared with the PND group in the PDA NP‐mediated NIR stimulation group (Figure 7N,P), but the oscillations of the other frequencies exhibited no significant changes (Figure 7O; Figure S23). Notably, the power of high‐gamma oscillations in the PDA NPs‐only group showed significant difference compared with the PND group. These results demonstrate that PDA NP‐mediated NIR stimulation improves cognition via enhancing hippocampal low‐ and high‐ gamma oscillations in PND mice.

## 3 Discussion

In this study, we explored transcranial non‐genetic neuromodulation via PDA NP‐mediated precise NIR stimulation. Melanin‐like PDA NPs were successfully prepared by the facile one‐step synthesis method. In vitro, PDA NP‐mediated 1064 nm NIR stimulation exhibited excellent photothermal efficacy, and also showed power‐dependent modulation effects on PC12 cells. In vivo, the photothermal effects increased the neuronal calcium activity as well as the gamma oscillations in the hippocampal DG region, thus improving cognitive function in naive and cognitive dysfunction mice. Our study suggests that PDA NP‐mediated 1064 nm NIR may be a potential strategy for enhancing cognitive functions.

Currently, precise and effective treatment for cognitive dysfunction is still difficult to achieve, and physical neuromodulation is gradually gaining attention as an emerging treatment [28]. Although transcranial electrical stimulation presents a potential cognitive enhancement, the accuracy of the stimulation position, especially for the deep brain nuclei, is limited, which can cause headache, dizziness, and other adverse reactions [29]. rTMS has been reported to be closely related to the occurrence of epilepsy during stimulation. Besides, some issues still persist, such as the inability to precisely target the treatment area, the risk of off‐target stimulation, and difficulty in repeatable positioning [30]. In contrast, transcranial NIR stimulation has a significant effect on cognitive enhancement with high biosafety. However, the low tissue penetration has a great limitation on its effectiveness on deep nuclei, while increasing the incident NIR power may lead to localized thermal damage on the irradiated site [31]. Therefore, introducing photothermal nanomediators into the target tissue to mediate NIR stimulation generates a photothermal effect in specific brain regions, achieving high localization accuracy. Additionally, the high penetration of 1064 nm NIR light enables more effective neuromodulation on the targeted region than other wavelengths of NIR.

The field of neuromodulation has witnessed significant progress driven by diverse energy transduction paradigms. The PDA NP‐mediated 1064 nm NIR photothermal neuromodulation system established in this study offers unique advantages. Compared to alternating magnetic field (AMF)‐dependent magnetothermal stimulation, this strategy circumvents the need for specialized magnetic‐sensitive metal nanoparticles, complex and extensive magnetic equipment, viral transfection and biological risks related to magnetic stimulation [32]. The melanin‐like nature of PDA NPs avoids the heavy metal toxicity and stability issues inherent in multi‐component systems, better aligning with clinical requirements for safety, simplicity, and long‐term tolerance. It should be noted, however, that unlike many magnetothermal strategies, our NIR approach necessitates the implantation of optical fibers on the skull surface to deliver light to deep brain regions [33]. This tethered configuration may, to some extent, restrict animal movement during experiments.

Recent pioneering studies by Jin et al. employed photoelectric nanoparticles to convert NIR light into visible light and subsequently into electrical signals, facilitating non‐genetic, fiber‐independent, and non‐invasive deep brain stimulation [13, 34]. In contrast, our strategy leverages the photothermal effect of PDA NPs to modulate neural activity. Specifically, we harness the photothermal properties of these natural melanin‐inspired PDA NPs to activate endogenous TRPV1 channels. Compared to complex multi‐component systems involving heavy‐metal‐based materials, PDA NPs may be superior due to their adhesive properties, the intrinsic radical scavenging capabilities and anti‐inflammatory effects, thereby enhancing their potential for clinical translation. Therefore, in the current precise NIR neuromodulation strategy, our research focuses more on the biosafety and long‐term tolerance, which are critical requirements for the treatment of chronic neurodegenerative diseases.

This study utilizes an extracellular photothermal strategy mediated by PDA NPs to modulate neuronal activity. Compared with intracellular neuromodulation (e.g., optogenetics or patch‐clamp–based modulation), with its millisecond temporal resolution and submillisecond precision [35], the response time of neurons to extracellular photothermal stimulation is usually in the range of hundreds of milliseconds to seconds [36], exhibiting lower temporal resolution. Nevertheless, extracellular photothermal stimulation also offers some distinct advantages. First, as a non‐genetic intervention, it eliminates the need for viral transfection, which is indispensable for optogenetics, thereby bypassing associated biosafety risks. Second, its minimally invasive nature prevents cumulative physical damage, facilitating long‐term and repeatable neuromodulation. Consequently, this biocompatible strategy serves as a robust complement to traditional intracellular methods, providing a safer avenue for investigating neural circuits and intervening in neurological disorders.

The molecular architecture of PDA NPs comprises a conjugated hybrid system of catechol, amine, quinone, and indole moieties, which endows them with a broad‐spectrum optical absorption spanning from the UV to the NIR regions. This allows for the highly efficient capture of 1064 nm NIR energy. Upon photon absorption, excited‐state electrons undergo non‐radiative relaxation, converting energy into lattice vibrational energy that is subsequently released as heat. The resulting photothermal conversion efficiency of PDA NPs is 30.4%, which significantly outperforms that of conventional similar photothermal materials. Moreover, PDA NPs exhibit superior intrinsic adhesion and neuro‐affinity, compared to other photothermal polymers such as polypyrrole and poly‐L‐DOPA [37]. Benefiting from the dense internal structure formed by strong π‐π stacking and dense hydrogen bonds, PDA NPs also display better structural stability in physiological environments than other photothermal polymers [38]. Furthermore, PDA NPs have an outstanding capacity for ROS scavenging and immunomodulation, which provides a synergistic neuroprotective effect [39]. These multifaceted attributes position PDA NPs as highly promising multifunctional mediators for neuromodulation with significant potential for clinical translation.

We selected PDA NPs with a diameter of 490 nm for NIR‐based neuromodulation. The reason is strategically based on an optimal balance between photothermal performance, physico‐optical effects, biological stability, and cellular interaction. First, our study and previous research both confirmed that the light absorption capacity of PDA NPs is positively correlated with their size; specifically, increasing the particle size from 299 to 426 nm resulted in a 141% surge in NIR absorbance, with the temperature elevation ∼40% higher than that of smaller counterparts [40]. Second, the 490 nm size satisfies the Mie scattering resonance condition for the 1064 nm laser, triggering internal field enhancement effects that significantly increase the effective optical absorption cross‐section. This serves as the core physical mechanism underlying their high‐efficiency photothermal conversion. Third, larger PDA NPs (>400 nm) have been reported to exhibit superior size retention, and a more compact, rigid structure, enhancing stability within physiological oxidative environments. Finally, 400–500 nm PDA NPs feature moderate cellular uptake efficiency that avoids rapid degradation associated with overly small particle sizes and increased cytotoxicity at high concentrations caused by excessively large particles (>900 nm), achieving a more balanced safety and efficacy profile for both in vitro and in vivo applications, making them well‐suited for long‐term neuromodulation applications.

Our findings demonstrate that PDA NPs exhibit long‐term retention within the target region of the hippocampal DG for at least three months post‐injection. This efficient local retention and restricted diffusion are primarily attributed to the large particle size and the inherent tissue adhesiveness of PDA NPs. Notably, the PDA NPs can degrade in situ, which is consistent with the degradation process of PDA NPs that we previously reported [41]. These properties facilitate the physical confinement of the nanoparticles within the extracellular matrix of the brain tissue. Such localized stability not only ensures the feasibility of chronic and reproducible neuromodulation but also guarantees high spatial precision during photothermal stimulation, effectively preventing off‐target effects. Collectively, these characteristics underscore the significant potential of the PDA NPs employed in this study as a safe and long‐lasting neuromodulatory mediator, facilitating prolonged therapeutic neuromodulation.

To further confirm that the neural regulation is indeed caused by the photothermal effect of PDA NPs rather than the physical presence effect of the nanoparticles themselves [42], we conducted additional experiments using SiO_2_ NPs with a similar particle size to PDA NPs, and found that SiO_2_ NP‐mediated 1064 nm NIR did not modulate cellular activity, and these data suggest that the photothermal effect produced by PDA NP‐mediated NIR stimulation is the main factor in the regulation of cell activity.

Temperature‐sensitive proteins play a key role in regulating cell behavior in response to external thermal stimuli [43]. TRPV1 forms a non‐selective cation channel that preferentially conducts mono‐ and divalent cations, with a notable preference for Calcium ion [44]. While TRPV1 can be activated when the temperature reaches or exceeds 42°C, this leads to the opening of the channel and the influx of calcium ions, thereby triggering an action potential [45]. Recently, Carmignani et al. utilized PDA NPs to activate cells through thermal activation under NIR light exposure in vitro, thereby triggering transient calcium ion fluctuations and other cellular responses [46]. Our research further confirmed in vivo that mild heating can cause calcium ion influx through the TRPV1 channel. However, we must acknowledge the inherent non‐specificity of heat as a physical stimulus. Various thermosensitive TRP channels, including TRPV3 and TRPM2, can be activated within the temperature range utilized in this study [47, 48, 49]. Although our pharmacological evidence strongly suggests that TRPV1 is the primary target, we cannot entirely rule out the possibility that other thermosensitive channels play a complementary or synergistic role in the observed calcium responses. Consequently, while we conclude that the PDA NP‐mediated photothermal effect triggers calcium activity primarily via TRPV1 activation, the precise contributions of other thermosensitive channels remain to be further elucidated in future studies using ion‐channel screening or gene‐knockout models.

For the in vitro experiments in this study, PC12 cells were employed as the neuronal model instead of primary neurons. Although PC12 cells exhibit certain physiological differences from primary neurons, they possess distinct neuron‐like characteristics and have long been established as a classic tool in neurobiological research [50]. Our results confirmed that the PC12 cell line used in this work robustly expresses TRPV1 (Figure S13), providing a necessary molecular foundation for our investigation. More importantly, previous studies have confirmed that activating the TRPV1 of PC12 cells with specific agents could efficiently induce calcium influx [51, 52]. Therefore, it is scientifically justifiable to use PC12 cells as an in vitro model to evaluate photothermal‐induced TRPV1 activation and the resulting changes in calcium activity.

Interestingly, PDA NP‐mediated NIR stimulation exhibited a power‐dependent modulation effect: while low‐power irradiation enhanced calcium signaling, high‐power stimulation led to a suppression of the calcium response. Conceptually, this inhibitory effect may be attributed to two primary mechanisms. First, thermal desensitization of TRPV1 likely serves as a critical factor. When exposed to persistent noxious heat, the N‐ and C‐termini of TRPV1 interact to drive pore domain rearrangement, leading to a selective desensitization that protects cells from swelling and tissue damage induced by excessive thermal stress [53]. Second, the activation of calcium homeostatic feedback may play a role. Excessive Calcium ion influx triggered by high‐power stimulation can activate negative feedback loops, such as calcium‐binding proteins or plasma membrane calcium pumps, to accelerate Calcium ion clearance and prevent excitotoxicity [47]. Our study focuses on the stable activation of the TRPV1 channel by PDA NPs within the physiologically safe temperature range. The power‐dependent modulation observed in vitro provides a conceptual framework for the future development of bimodal excitatory‐inhibitory neuromodulation strategies. However, whether these effects can be faithfully replicated within complex in vivo neural networks warrants further investigation.

To ensure technical stability during probe insertion and thermographic monitoring, absolute temperature measurements were recorded in anesthetized mice. However, anesthesia significantly suppresses metabolic rate and thermoregulation, leading to a lower baseline brain temperature. In contrast, previous literature has reported that the baseline brain temperature of awake, freely moving mice is consistently higher, typically ranging from 37.0°C to 38.0°C [54]. Based on our measurements, the ΔT achieved at target power was 3.4°C. Therefore, the absolute brain temperature in awake mice during NIR stimulation is estimated to reach 41.4°C. However, emerging evidence suggest that the TRPV1 activation threshold is not a fixed value of 42°C in vivo; a rise from 37°C to 39°C is sufficient to partially gate TRPV1 channels [18, 55], resulting in a 2.3‐fold increase in whole‐cell TRPV1 conductance. Thus, even with a conservative estimate, our photothermal strategy provides sufficient thermal energy to gate these channels and modulate neuronal activity.

It should also be noted that measuring the temperature within living organisms remains a challenge, and there is inconsistency in the absolute values of the temperature increases measured through simulation and experiments. In the in vivo experiments, temperature changes were measured using an invasive thermocouple probe. Previous studies have noted that contact‐based thermometry, including thermocouples, requires insertion of the sensing element into tissue and provides localized single‐point measurements rather than a complete spatial temperature map [56, 57]. Moreover, the probe itself may perturb the local thermal field; temperature probes or catheters can absorb energy more rapidly than surrounding tissue, leading to readings higher than the undisturbed tissue temperature, and localized hot spots may form near thermocouple probes under certain heating conditions. Therefore, the simulated temperature rise should not be interpreted as an exact reproduction of the thermocouple‐measured temperature, but rather as a model‐derived estimate for evaluating the spatial distribution pattern and relative enhancement of photothermal effects under different conditions. Future studies will incorporate non‐invasive or minimally perturbative temperature measurement approaches to further validate the true thermal response of the system.

Neuronal activity is tightly coupled to energy metabolism. Recently, studies have revealed that learning, memory, or artificially induced neural activity significantly enhances mitochondrial gene transcription near neuronal synapses, thereby promoting energy supply to the brain. Further research has demonstrated that the coupling between neural activity and mitochondrial gene expression is highly dependent on the influx of mitochondrial calcium ions induced by neural activity [58, 59]. Accordingly, PDA NP‐mediated NIR stimulation may improve cognitive function in PND mice by stimulating hippocampal neuron activity, thereby enhancing mitochondrial membrane potential and increasing ATP production.

Although NIR irradiation alone can enhance metabolism and improve cognitive function by activating cytochrome c oxidase (CCO) [60, 61], in this study, it did not show significant improvements in cognitive function, whether in naive mice or those with cognitive dysfunction. This is likely because the low power density of the NIR light used in the current study was insufficient to generate the ATP levels required to significantly enhance cognition. This finding aligns with our in vitro results, where no significant increase in ATP content was observed in the NIR‐only group compared to the control group.

Oscillatory activity in the brain plays a crucial role in the temporal organization of information transfer and the neural activity patterns within large‐scale brain networks involved in learning and memory‐related behaviors [62, 63, 64]. Gamma oscillations (30‐120 Hz) synchronize neuronal activity linked to cognitive processes, and their disruption is observed in many neurodegenerative diseases, such as Alzheimer's disease (AD) [65, 66]. Research indicates that gamma oscillations are primarily driven by inhibitory networks centered on parvalbumin (PV)‐positive interneurons. PV neurons are characterized by extremely high firing rates and metabolic demands that significantly exceed those of ordinary pyramidal neurons, rendering them highly sensitive to fluctuations in intracellular ATP levels [67, 68, 69, 70]. In disorders associated with cognitive impairment, insufficient ATP supply resulting from mitochondrial dysfunction often preferentially impairs the function of PV neurons, subsequently leading to attenuated gamma oscillations and cognitive deficits. Consequently, the significant increase in gamma oscillation power observed in mice in this study may be attributed to enhanced energy metabolism, which provides sufficient ATP to the PV neuron network. This likely enabled the restoration of high‐frequency synchronized firing, thereby optimizing information processing and memory encoding efficiency within the hippocampus, ultimately manifesting as improved spatial memory performance in the Barnes maze. However, further investigation is required to comprehensively understand the specific impacts of PDA NP‐mediated photothermal effects on neuronal firing patterns and broader neural network dynamics.

Under low‐power NIR irradiation, a marginal decrease in supernatant DA concentration was observed, likely due to heat‐accelerated adsorption or oxidative polymerization of residual monomers. This confirms that the parameters used for cognitive enhancement do not induce PDA NPs degradation or DA release. Although high‐intensity NIR caused a slight DA increase, such settings were avoided in vivo to prevent overheating. Crucially, the instantaneous neural activation aligns with the rapid kinetics of photothermal effects rather than the slower temporal dynamics of DA receptor‐mediated signaling. These results collectively demonstrate that the observed neuromodulation is driven by a photothermal mechanism rather than chemical signaling.

Notably, the group treated with PDA NPs alone also exhibited modest cognitive improvement in the PND model. Given that oxidative stress is a key pathological mechanism of PND, this protective effect may be attributed to the abundance of phenolic hydroxyl and amine groups in PDA NPs. These functional groups endow the nanoparticles with intrinsic ROS scavenging capabilities [71], which inhibit M1 microglial polarization and reduce the release of pro‐inflammatory cytokines, thereby exerting antioxidant and anti‐neuroinflammatory effects [72]. However, this hypothesis warrants further experimental validation.

Despite the advantages of this approach, the requirement for local intracranial injection precludes this strategy from being entirely non‐invasive, potentially hindering its widespread clinical adoption. Future research will explore the functionalization of nanoparticles with targeting peptides or the use of focused ultrasound‐induced blood‐brain barrier opening to achieve truly non‐invasive and precise neuromodulation. Meanwhile, we acknowledge that systematically evaluating the size‐dependent functional differences of PDA NPs at the organismal level will be essential for fully ascertaining their potential for in vivo applications. Consequently, this represents a key direction for our upcoming investigations. Moreover, further investigation into the mechanisms of PDA NP‐mediated NIR modulation on neuronal activity and cognitive improvement is essential for advancing its clinical translation.

In summary, our study explored a transcranial neuromodulation strategy via PDA NP‐mediated precise 1064 nm NIR stimulation for cognitive enhancement in mice. Benefiting from the excellent photothermal properties of PDA NPs, this approach displayed controlled power‐dependent regulation of neuronal activity in vitro. The improved cognitive function in mice driven by this photothermal effect by enhancing neuronal activity and promoting gamma oscillations in the hippocampal DG region. Thus, this neuromodulation approach may hold promise as a novel strategy for the future treatment of cognitive dysfunction.

## 4 Experimental Section

### 4.1 Animals

All experimental procedures were approved by the Ethics Committee of Zhengzhou University. Adult male C57BL/6J mice (8–12 weeks old) were purchased from Beijing SiPeiFu Biotechnology Co Ltd. (Beijing, China). Five animals were housed per cage in standard laboratory conditions (12‐h light/dark cycle, lights on from 8:00 a.m. to 8:00 p.m.) and temperature (23–24°C) with free access to food and water. All animal experiments were carried out in line with the National Institutes of Health guide for the care and use of laboratory animals.

### 4.2 Synthesis of PDA NPs

PDA NPs were synthesized via the precisely controlled oxidative self‐polymerization of dopamine hydrochloride in an alkaline aqueous solution to achieve effective regulation of particle size. Briefly, 90 mL of ultrapure water (UPW), 40 mL of anhydrous ethanol, and 0.75 mL of aqueous ammonia were sequentially added to a round‐bottom flask. The mixture was magnetically stirred at room temperature for 30 min to form a homogeneous alkaline solution. Subsequently, 500 mg of dopamine hydrochloride powder was dissolved in 10 mL of UPW and introduced into the stirring alkaline mixture. The reaction was maintained under continuous stirring at room temperature for 24 h. Upon completion, the reaction mixture was transferred to centrifuge tubes and centrifuged at 15,000 rpm for 15 min to collect the nanoparticles. The supernatant, containing unreacted dopamine monomers and small oligomers, was discarded. The resulting pellet was resuspended in UPW and centrifuged again. This purification process was repeated until the supernatant became clear and transparent. The purified PDA NPs were finally redispersed in UPW and stored at 4°C, protected from light. During these procedures, the size of the obtained PDA NPs could be adjusted by the concentration of dopamine hydrochloride and aqueous ammonia as well as the reaction temperature and time.

### 4.3 Calculation of Photothermal Conversion Efficiency (PCE)

The photothermal conversion efficiency (η) of the PDA NPs was determined according to the energy balance model reported by Roper et al. using the following equation [73]:

(1) $$\eta =\frac{\mathrm{hA}\left(T_{\max}-T_{\mathrm{surr}}\right)-Q_{0}}{I\left(1-10^{-A_{\lambda }}\right)}$$

where T_max_ and T_surr_ are the maximum equilibrium and ambient temperatures, respectively, I is the incident laser power, A_λ_ is the absorbance of the PDA NPs dispersion at 1064 nm, and Q_0_ is the heat baseline of the solvent and cuvette measured independently.

The heat transfer coefficient term (hA) was derived from the cooling profile after laser extinction using the relationship hA = mC_p_/τ_s_, where m is the dispersion mass, C_p_ is the specific heat capacity of water (4.18 J·g^−^^1^·C^−^^1^), and τ_s_ is the system time constant. τs was obtained by linearly fitting the cooling time (t) against −ln(θ), where θ is a dimensionless temperature parameter defined as (T−T_surr_)/(T_max_−T_surr_).

### 4.4 Photothermal Stimulation of PC12 Cells

PC12 cells were incubated with PDA NPs in 6‐well plates for 24 h. Then cells were washed with PBS three times and stained with 5 µM of Fluo‐4 AM diluted in HBSS for 20 min. Fivefold volume of HBSS containing 1% FBS was added to incubate for another 40 min. Afterward, HEPES was used to wash and incubate the cells. A 1064‐nm NIR laser was incorporated into the inverted fluorescence microscope system.

### 4.5 Photothermal Stimulation of Hippocampal Neurons in Vivo

Mice were anesthetized by 2.5% isoflurane during the surgery. Their temperature was maintained by a warm pad. Then, PDA NPs were stereotactically injected into the hippocampus of mice (AP: −1.8 mm, ML: ±1.3 mm, DV: −1.9 mm). A 1064 nm NIR laser is emitted through the cannula, and the temperature of the hippocampal region is measured in real time through the administration cannula.

### 4.6 In Vitro Cytotoxicity Assay

Cells were seeded at a density of 5 × 10^3^ /well on 96‐well plates and incubated in culture medium for 24 h. Afterward, cells were treated with PDA NPs with variable concentrations for 24 h. Then, CCK‐8 solution was added into the DMEM medium to evaluate the cell viability. The effects of NIR irradiation on cell viability were also tested. After 24 h of attachment, cells were treated with PDA NPs for 12 h and then irradiated by 1064 nm laser for 5 min. After re‐incubation for another 12 h, cell viability was evaluated by CCK‐8 assay.

### 4.7 Detection of Mitochondrial Membrane Potential

Aspirate the medium from 35 mm cell culture dish, wash the cells once using PBS, add 1 mL of medium, add 1 mL of JC‐1 staining working solution and mix thoroughly. The cells were incubated in a cell culture incubator at 37°C for 20 min. At the end of incubation at 37°C, the supernatant was aspirated, washed twice with JC‐1 staining buffer, and observed under a fluorescence microscope after adding 2 mL of cell culture solution.

### 4.8 Detection of ATP Levels

Melt the reagent to be used on an ice bath and dilute the ATP standard solution with ATP assay lysate to an appropriate concentration gradient. Take appropriate amount of ATP assay reagent and dilute ATP assay reagent with ATP assay reagent diluent at the ratio of 1: 9. Aspirate the medium from 35 mm culture dish, add 200 µL of lysate per dish, use a pipette to perform repeated blowing or shake the culture plate to make the lysate fully contact and lyse the cells, centrifuge the cells at 4°C, 12000 g for 5 min after lysis, and take the supernatant. Add 100 µL of ATP assay working solution to the assay wells and leave it at room temperature for 3‐5 min to allow all the background ATP to be consumed, thus lowering the background, then add 20 µL of sample or standard to the assay wells, mix quickly with a micropipette, and after a 2‐s interval, assay with a luminometer.

### 4.9 Detection of Dopamine Content

Dopamine (DA) levels in samples were determined using a fluorometric Dopamine Assay Kit (S0580, Beyotime Biotechnology, Shanghai, China). A standard curve was generated using a gradient of DA concentrations ranging from 0 to 100 µM, prepared from a 1 mM DA stock solution. For the assay, 20 µL of sample or standard was added per well, followed by the sequential addition of 30 µL of DA Probe and 50 µL of DA Assay Buffer. The reaction was incubated at 25°C in the dark for 30 min. Fluorescence intensity was measured at an excitation wavelength of 417 nm and an emission wavelength of 460 nm using a microplate reader. Dopamine concentrations were calculated based on the standard curve and corrected for the dilution factor.

### 4.10 Surgery

The surgery was a left carotid artery exposure. Briefly, mice were anesthetized with 2.5% isoflurane delivered using an agent‐specific vaporizer in a 30% oxygen and air mixture for 10 min before surgery. A 2.0 cm long incision was made along the midline of the neck, and 1 cm of the left carotid artery was carefully dissected from the surrounding tissues, avoiding damage to the vagus nerve. The wound was sutured with sterile surgical sutures. Sterile conditions were ensured throughout the 15‐min surgical procedure.

### 4.11 Y Maze Test

The Y maze spontaneous alternation test measures exploratory behavior based on the willingness of the mice to visit a new arm of the maze rather than a familiar arm. The apparatus used consisted of three enclosed arms with an angle of 120 degrees; each arm was 30 cm in length, 8 cm in width, and 15 cm in height. Each mouse was gently placed in the center of the maze and traced by a video‐tracking system for an 8‐min period in a quiet, dimly lit (30 lux) room. The sequence and number of arm entries were then scored by a person who was blinded to the experiment. A spontaneous alternation was defined as a mouse entering all three arms on consecutive choices (ABC, BCA, or CAB but not BAB or CAC or CBC). The percentage of spontaneous alternation was calculated as shown in the following equation: % spontaneous alternation = number of spontaneous alternations / (total arm entries − 2) * 100.

### 4.12 Barnes Maze Test

Mice were subjected to the Barnes maze test to assess spatial learning and memory. The mouse was placed in the center of a circular platform with 20 equally spaced holes. One of the holes was connected to a dark chamber called target hole. Aversive noise (85 dB) and bright light (200 W) shed on the platform were used to encourage the mouse to find the target hole. The spatial acquisition training phase was 5 days, 4 min per trial. The spatial memory tests were performed on the first day after the training phase. The latency to find the target hole was recorded by the Super‐maze video tracking system.

### 4.13 Immunohistochemistry

Mice were deeply anesthetized with isoflurane and slowly perfused with phosphate‐buffered saline (PBS) and 4% paraformaldehyde (PFA). Brains were carefully removed and postfixed in 4% PFA overnight, then dehydrated with 20% and 30% sugar in PBS and coronally cut into 25 µm‐thick brain slices using a cryostat (CM1950, Leica). The sections were blocked with 5% normal goat serum and 0.3% Triton X‐100 in PBS for 1 h at room temperature followed by incubation with primary antibody overnight at 4°C. The following primary antibodies were used: rabbit anti‐Iba1 (1:1,000, Wako), rabbit anti‐GFAP (1:200, CST), and rabbit anti‐c‐Fos (1:300, CST). Next, wash the sections three times with PBS before incubating them with the goat anti‐rabbit IgG‐Cy3 (1:400, 111165003, Jackson ImmunoResearch) for 2 h at room temperature. Finally, 4',6‐diamidino‐2‐phenyl‐indole (DAPI) was used to immerse the slices for 5 min for nuclear staining for localization. Fluorescence microscopy images were collected by a confocal scanning microscope (A1 MP+, Nikon).

### 4.14 In Vivo Temperature Recording

After anesthetizing the mice with tribromoethanol and fixing them on the adapter of the stereotaxic instrument, a micro‐dosing cannula (RWD, Shenzhen, China) was implanted into the dentate gyrus of the mouse hippocampus. The cannula was fixed to the mouse skull using glass‐ionomer cement. After the surgery, the mice were isolated and allowed to recover for 2 days. During the test, the mice were anesthetized with a face mask inhalation of isoflurane to maintain the anesthesia state. The mice were fixed on the adapter of the brain stereotaxic instrument, and a T‐type ultrafine thermocouple (0.05 mm, KAIPUSEN, China) was inserted into the micro‐dosing cannula and connected to a thermocouple temperature measuring instrument for temperature recording. Once the temperature in the mouse brain stabilized, the temperature in the brain was recorded.

### 4.15 In Vivo Electrophysiological Recording

A 4 × 2 electrode array that consisted of 8 microwires (25 µm in diameter, Kedou, Suzhou, China) was implanted into the DG. Two screws were fixed on the skull and attached to stainless steel wires as ground. After surgery, the mice were kept separately and allowed to recover for 7 days. All mice were habituated to the recording homestage for 10 min per day for 2–3 days in the test environment. Mice were placed in a square box wrapped with a copper mesh (freely moving test) or an insulated plastic net wrapped with a copper mesh. In vivo signals were recorded by an Apollo II 32ch digital signal processor (Bio‐Signal Technologies, Nanjing, China), amplified, filtered at a 300–5000 Hz bandwidth and stored by Apollo II 32ch acquisition software. The data were analyzed by an Offline Sorter (Plexon, USA) and Neuroexplorer (Nex Technologies, USA). We classified well‐isolated units using an unsupervised clustering algorithm based on a κ‐means method. The spikes were sorted offline based on three principal components (half‐spike width, half‐valley width and mean firing rate). Z‐score comparisons were analyzed using the averaged Z‐score values calculated within 1 s before and after laser on.

### 4.16 Fiber Photometry Recording

For fiber photometry recording, an optical fiber (200 µm OD, China, Inper Ltd.) was implanted 100‐200 µm above the viral expressed terminal site in the DG 2 weeks after viral injection. The mice were kept separately and allowed to recover for 7 days after surgery. Calcium signals were recorded with a fiber photometry system (China, Inper Ltd.). Calcium‐dependent signals were detected with a 470 nm LED (45 mW), and calcium‐independent signals were detected with a 410 nm LED (30 mW) as an isosbestic control. We segmented the data based on the laser on within the individual trials. The values of fluorescence change (ΔF/F) were obtained by calculating (F—F0)/F0, and the ΔF/F of 2 s before stimulus presentation was defined as the baseline. The photometry data were analyzed using InperPlot software (China, Inper Ltd.).

### 4.17 Photothermal Modeling and Monte Carlo Simulation of Mouse Brain

To model tissue heating under tNIR illumination, we performed optical Monte Carlo simulations using the pmcx and simulated the spatio‐temporal temperature changes of the brain tissue using the Pennes Bioheat Transfer Equation (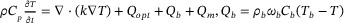) [74]. The optical heat source *Q_opt_* was obtained from the light energy distribution simulated with our MC model and the effective photothermal conversion coefficient (η), for the PDA NPs region, η was set to 0.4 based on previous measurements and for native brain tissue, η is close to 1. *Q_b_* is the blood perfusion, *Q_m_* is the metabolic heat generation, ρ_*b*_ is the blood density, ω_*b*_ is the blood perfusion, *C_b_* is the blood specific heat capacity, and *T_b_* is the blood temperature. The initial temperature was set to 37°C for the entire domain. For the optical Monte Carlo simulations, mouse model was segmented into six tissue types: gray matter, white matter, cerebrospinal fluid, skull, scalp, and a PDA‐injected region. Tissue labels were assigned based on probability maps (TPMs). The PDA NP‐injected region was approximated as a 1‐mm‐diameter spherical region within the segmented brain volume, representing a simplified model of the local PDA distribution after stereotactic injection. Its center was positioned at a dorsoventral depth of 1.9 mm, matching the reported in vivo injection depth (DV = −1.9 mm). To constrain the model variables and avoid introducing additional uncertain parameters, the local injection of PDA NPs was modeled as an enhancement of the effective photothermal absorption capability within the injected region, while the intrinsic thermal and bioheat parameters of the surrounding tissue were assumed to remain unchanged in the current model. A point source launched 1×10^8^ photons along the Z‐axis and the output was the voxel‐wise deposited energy, Q (J per voxel), calculated by scaling the normalized Monte Carlo energy deposition with the total optical energy emitted from the light source. For each 1s interval, the emitted energy was set to 0.03 J, corresponding to the irradiance used in the in vivo experiment. The heat source within a unit voxel can be calculated as *Q* · η. Bioheat transfer properties are summarized in Table S2.

### 4.18 Statistical Analysis

Animals were randomly assigned to experimental groups using a random number generator. To ensure objectivity, a double‐blind protocol was implemented: personnel involved in data acquisition and analysis remained blinded to the experimental groups until the final analysis. Inclusion and exclusion criteria: Data were included for analysis only if they met the following pre‐established criteria: (i) histological confirmation of correct electrode or optical fiber placement; (ii) precise verification of PDA NPs injection sites; and (iii) a signal‐to‐noise ratio sufficient for reliable data extraction. Animals exhibiting severe physiological stress or health complications were excluded from the study.

## Author Contributions

**Qiong Xue**: methodology, formal analysis, data curation. **Yan‐Bo Zhou**: conceptualization, writing – original draft, visualization, formal analysis, data curation. **Daqing Ma**: writing – review and editing. **Pan‐Miao Liu**: writing – original draft, writing – review and editing, conceptualization, supervision, funding acquisition, project administration, resources. **Ting‐Ting Zeng**: visualization. **Jian‐Jun Yang**: conceptualization, supervision, writing – review and editing, funding acquisition, project administration, resources. **Ke‐Yao Zhang**: investigation. **Wei‐ Tong Pan**: investigation. **Yu‐Ge Wang**: visualization. **Su‐Xuan Hou**: investigation. **Chenguang Zhao**: methodology, validation.

## Funding

National Natural Science Foundation of China grant U23A20421, 82371207, 82401421. Natural Science Foundation of Henan grant 242300421082. The Scientific Research and Innovation Team of The First Affiliated Hospital of Zhengzhou University grant ZYCXTD2023012.

## Conflicts of Interest

The authors declare no conflicts of interest.

## Supporting information




**Supporting File**: advs76611‐sup‐0001‐SuppMat.docx.

## Data Availability

The data that support the findings of this study are available from the corresponding author upon reasonable request.
