# Supplementary material for: Polydopamine Nanoparticle‐Mediated Precise Near‐Infrared Optical Stimulation for Cognitive Enhancement
Source: Adv Sci (Weinh). 2026 Jul 28:e76611. Online ahead of print. doi: 10.1002/advs.76611 (PMC13410804; doi:10.1002/advs.76611)
Supplement: Supplementary file 1 — Supporting File: advs76611‐sup‐0001‐SuppMat.docx. [file ADVS-9999-e76611-s001.docx]

**Supporting Information**

**Polydopamine Nanoparticle-Mediated Precise Near-Infrared Optical Stimulation for Cognitive Enhancement**

*Yan-Bo Zhou, Qiong Xue, Su-Xuan Hou, Ke-Yao Zhang, Wei-Tong Pan, Ting-Ting Zeng, Yu-Ge Wang, Daqing Ma, Chenguang Zhao*, Pan-Miao Liu*, Jian-Jun Yang**

**Additional details of photothermal modeling and Monte Carlo simulation of the mouse brain:**

The tissue temperature increase induced by transcranial near-infrared (tNIR) irradiation involves a sequence of physical processes, including photon propagation, optical absorption, photothermal conversion, and thermal diffusion. In the simulation model, optical Monte Carlo simulation was first performed to estimate the spatial distribution of light deposition in the mouse head, and the resulting optical heat source was then coupled with the Pennes bioheat transfer equation to simulate the spatiotemporal temperature evolution.

The anatomical model was segmented into six tissue types, including background air, gray matter, white matter, cerebrospinal fluid, skull, and scalp. Gray matter, white matter, and cerebrospinal fluid were assigned based on tissue probability maps, and outer skull and scalp layers were generated using morphological operations. The PDA NP-containing region was modeled as a spherical ROI with a diameter of 1 mm, centered near the experimentally targeted hippocampal DG region at a dorsoventral depth of approximately 1.9 mm. Each tissue type was assigned optical parameters at 1064 nm, including the absorption coefficient *μ_a_*, scattering coefficient *μ_s_*, anisotropy factor *g*, and refractive index (Table S1). PDA NPs injection was represented by increasing the optical absorption coefficient *μ_a_* within the defined injection ROI, while the intrinsic thermal properties of the surrounding tissue were kept unchanged.

The light source was modeled as a uniform circular disk beam with a diameter of 600 μm, incident along the Z-axis. A total of 1×10^8^ photons were launched per simulation, and their stochastic interactions with tissue boundaries—including absorption, scattering, and reflection—were tracked using GPU-accelerated Monte Carlo simulation. Energy deposition in tissue was modeled using the Beer–Lambert law, which accounts for exponential decay of photon intensity along the propagation path.


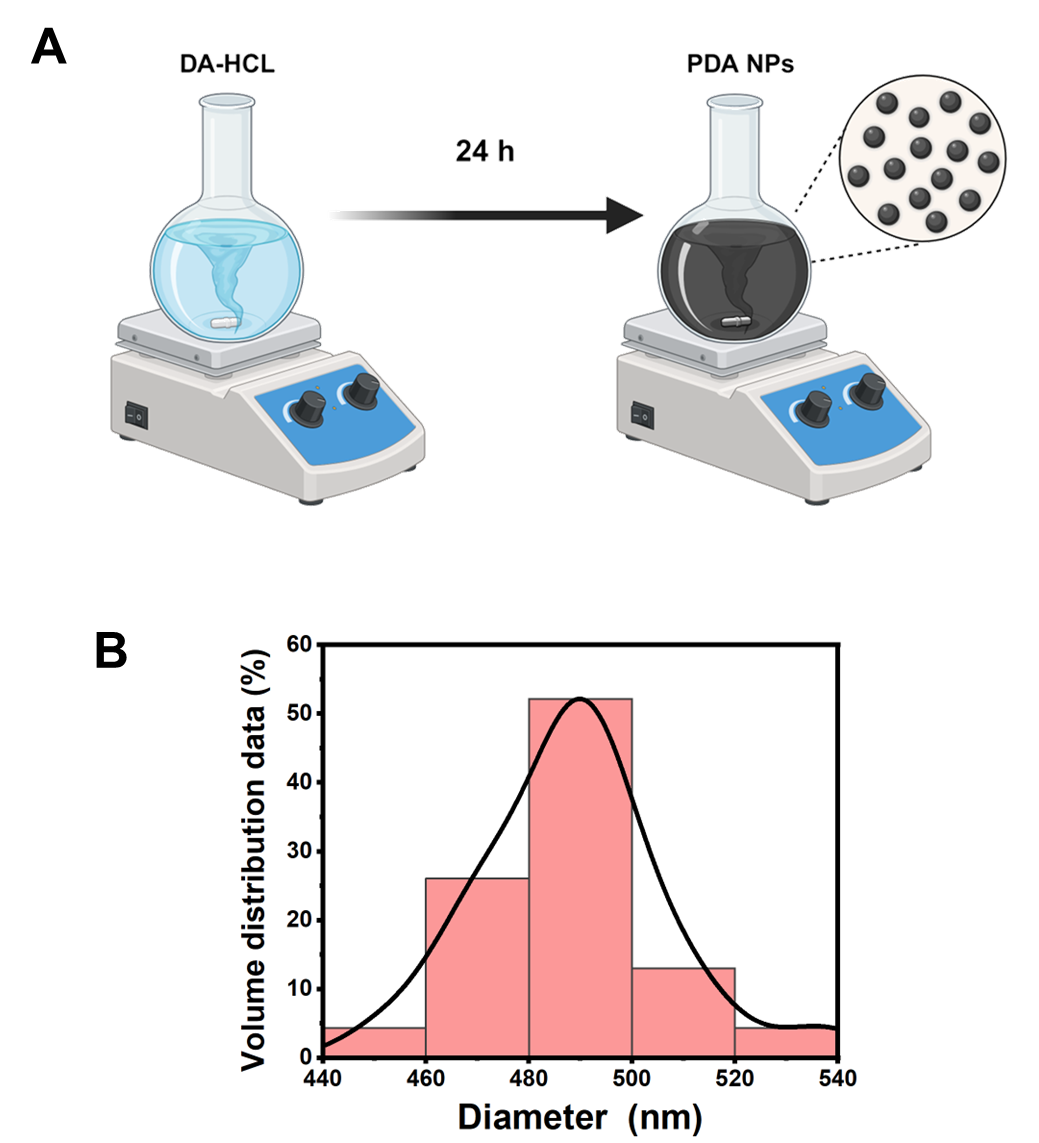


**Figure S1.** **(A)** Schematic of the preparation of PDA NPs. **(B)** Particle size distribution histogram of PDA NPs.


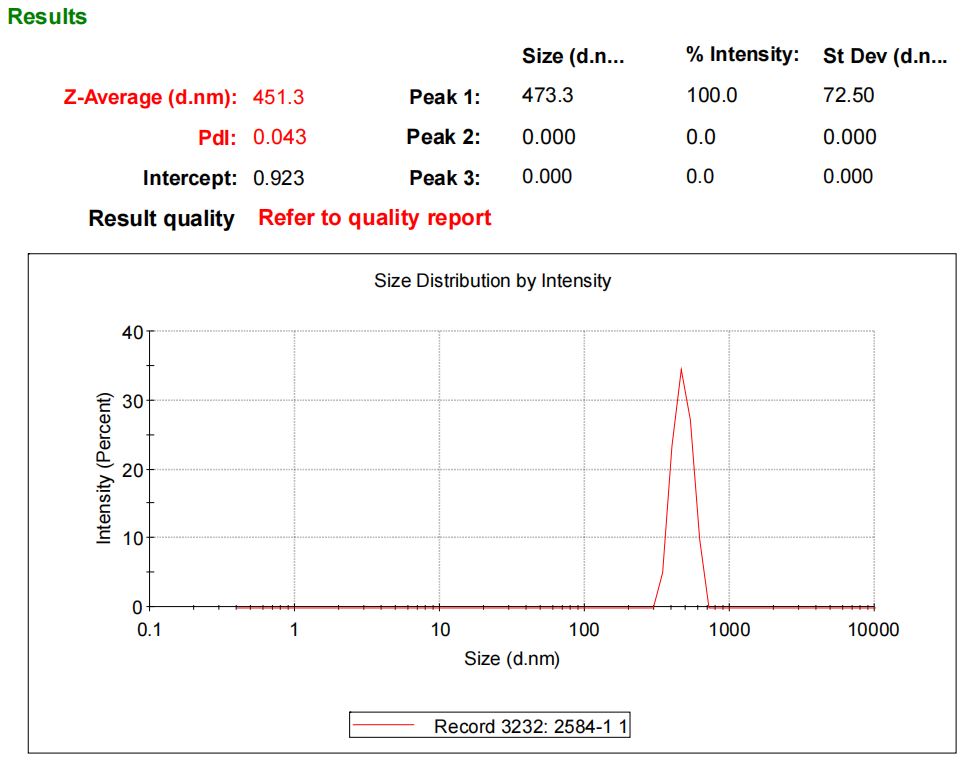


**Figure S2.** **The dynamic light scattering (DLS) results of the PDA NPs.**


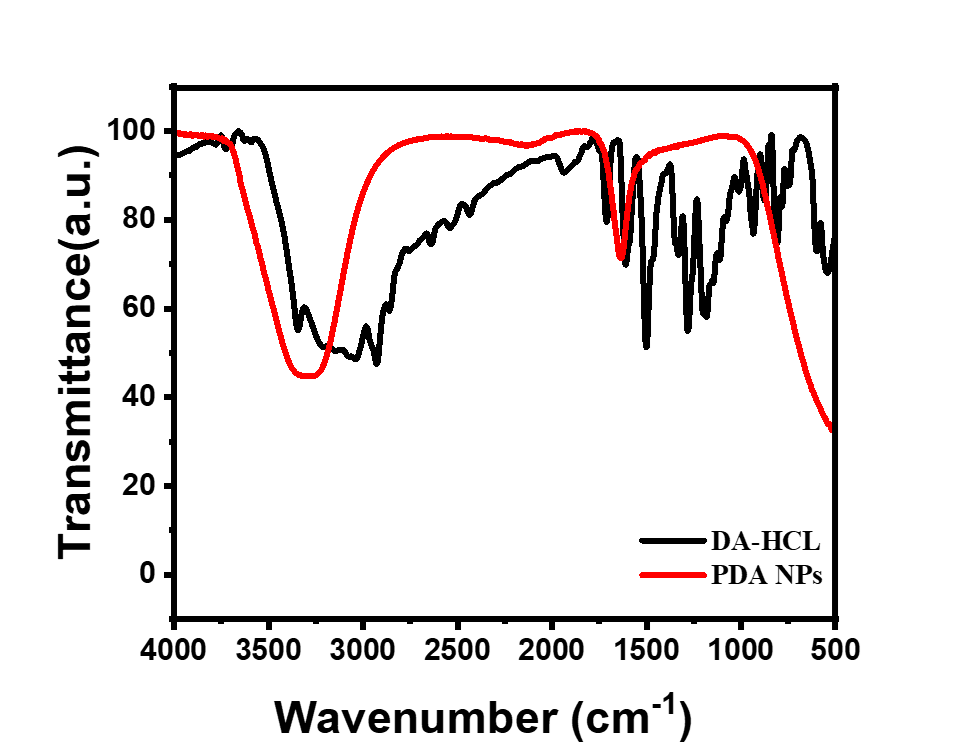


**Figure S3.** **FT‑IR spectra of DA-HCl and PDA NPs.**

**Figure S4. Stability testing of PDA NPs in various physiological-related media.** PDA NPs dispersed in H_2_O solution, PBS solution, MEM solution, and 10% FBS solution. PBS: Phosphate Buffered Saline, MEM: Minimum Essential Medium, FBS: Fetal Bovine Serum.


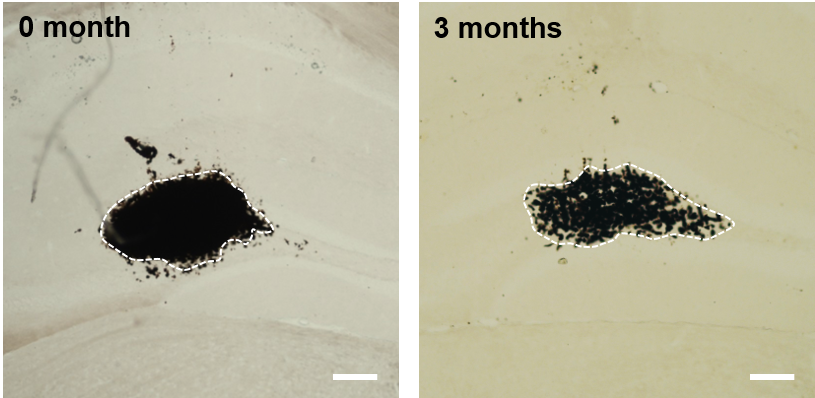


**Figure S5. The stability of PDA NPs in vivo.** Pictures of PDA NPs injected into the hippocampal brain region three month later. Scale bar,100 μm.


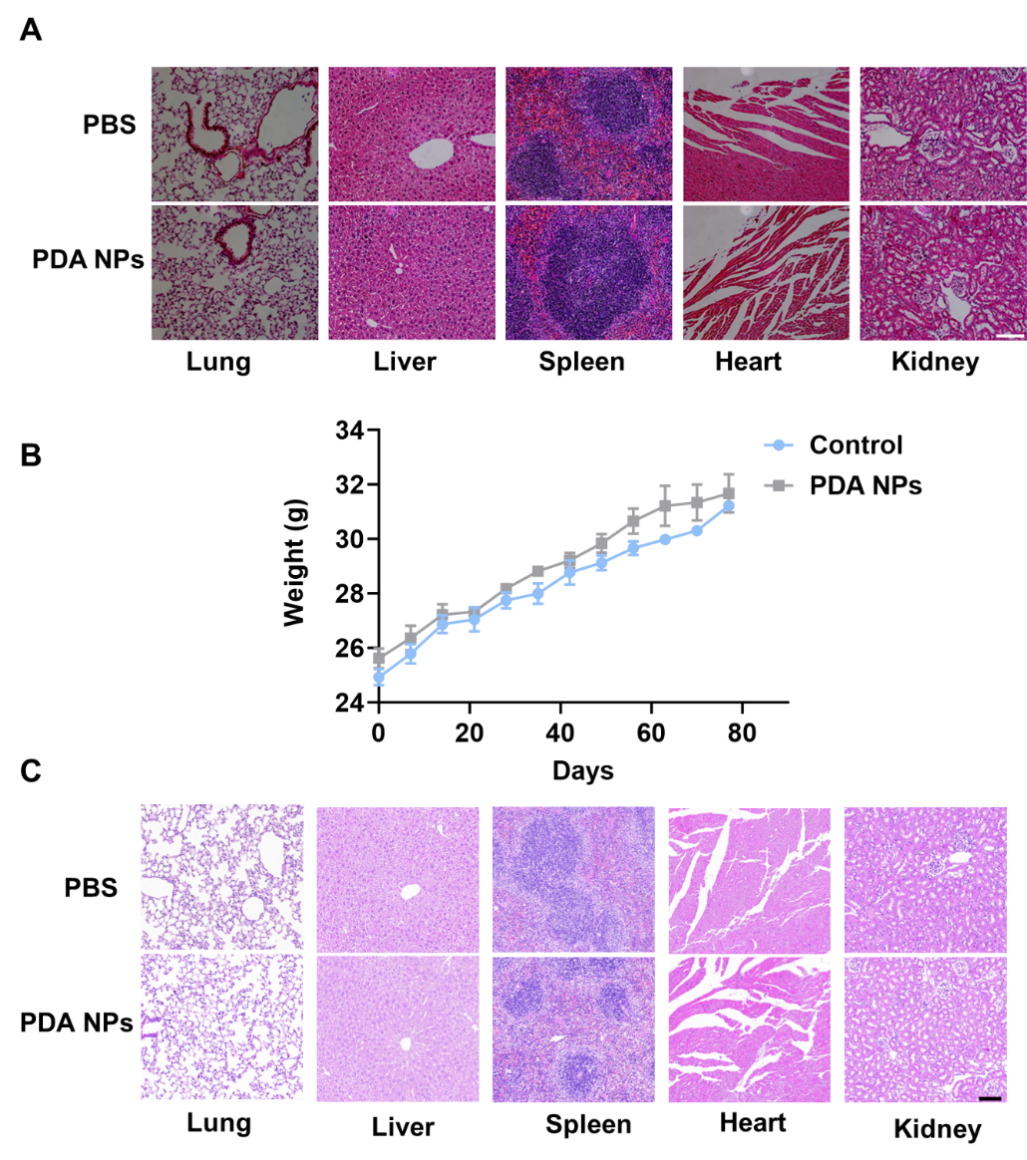


**Figure S6. (A)** Representative histological images of major organs at 1 week post-treatment. Scale bar, 100 μm. **(B)** The body weight changes of mice after PDA NPs injection. **(C)** Representative histological images of major organs at 3 months post-treatment. Scale bar, 100 μm.


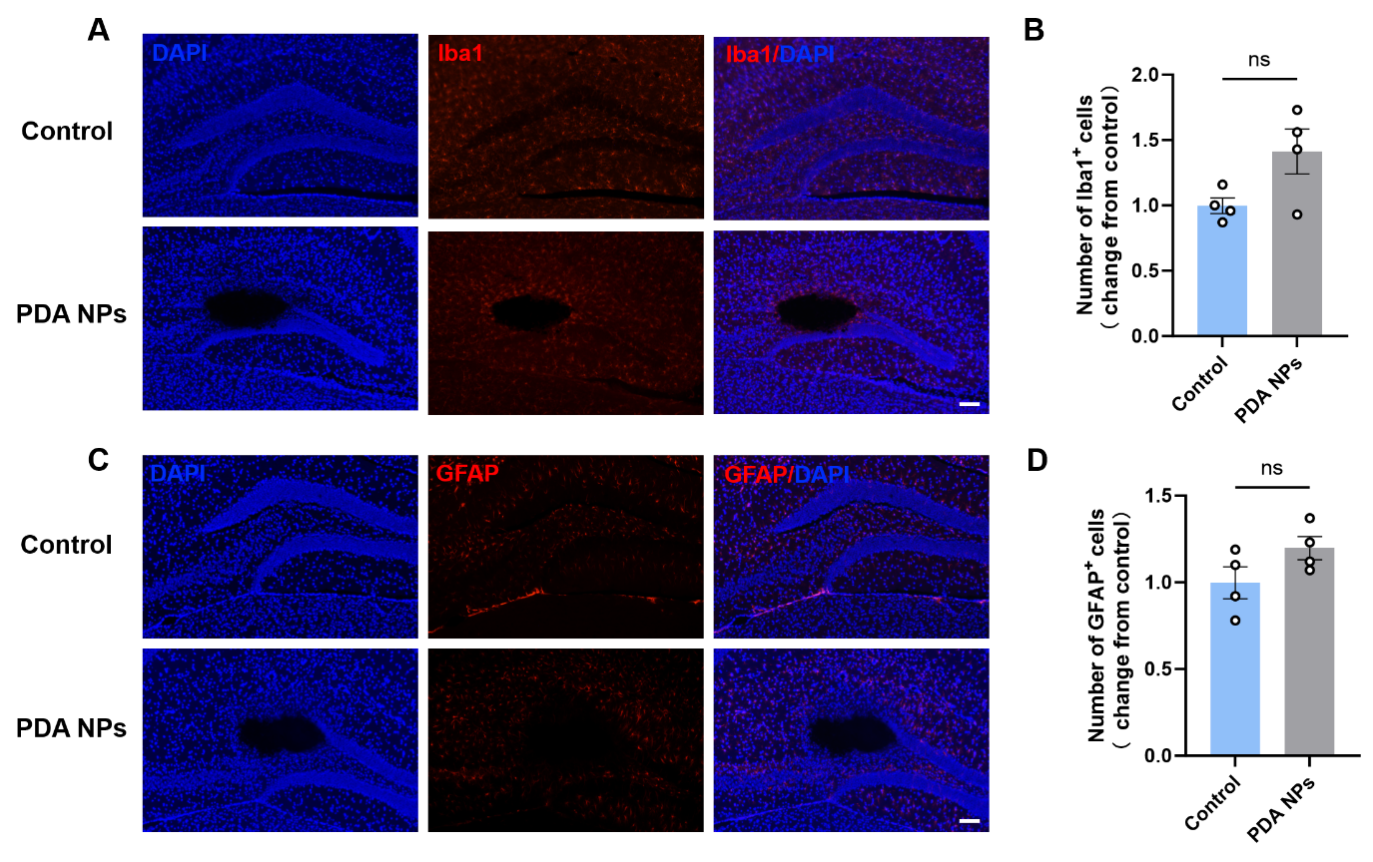


**Figure S7. Assessment of the chronic immune response in the mouse brain due to PDA NPs injection. (A)** Representative immunofluorescence of Iba1 after PDA NPs injection in DG region. Scale bar,100 μm. **(B)** Number of Iba1^+^ cells in control and PDA NPs groups. (t_(6)_ =2.274, p =0.0633), values are shown as mean ± SEM (n=4 mice/group). **(C)** Representative immunofluorescence of GFAP after PDA NPs injection in DG region. Scale bar,100 μm. **(D)** Number of GFAP^+^ cells in control and PDA NPs groups. (t_(6)_ =1.766, p =0.1279), values are shown as mean ± SEM (n=4 mice/group). B, D) Results were analyzed by unpaired student's t- test.


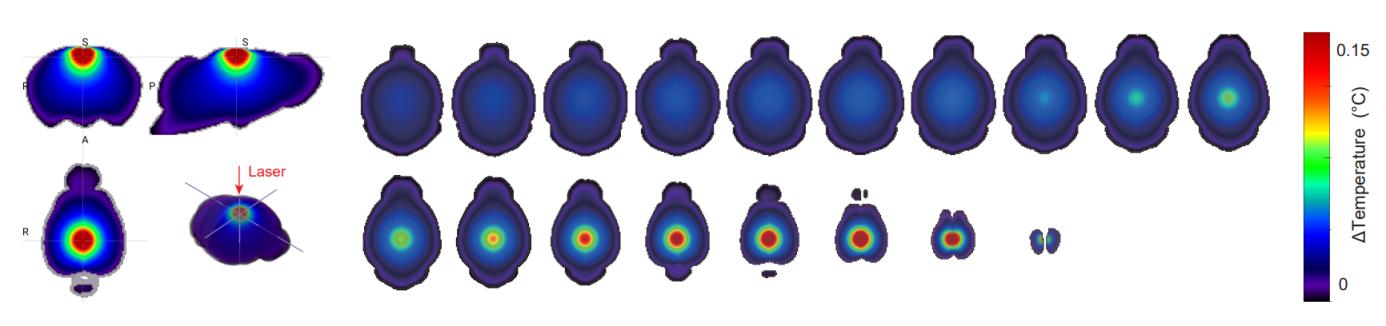


**Figure S8.** Simulated 3D temperature rise distributions in the mouse brain without PDA NPs injection, showing laser-induced heating under transcranial illumination. The figure includes three orthogonal slices and a semi-transparent 3D view with the skull partially removed. The red arrow indicates the laser incident direction along the Z-axis. Axial temperature maps show the spatial thermal distribution across successive brain slices.


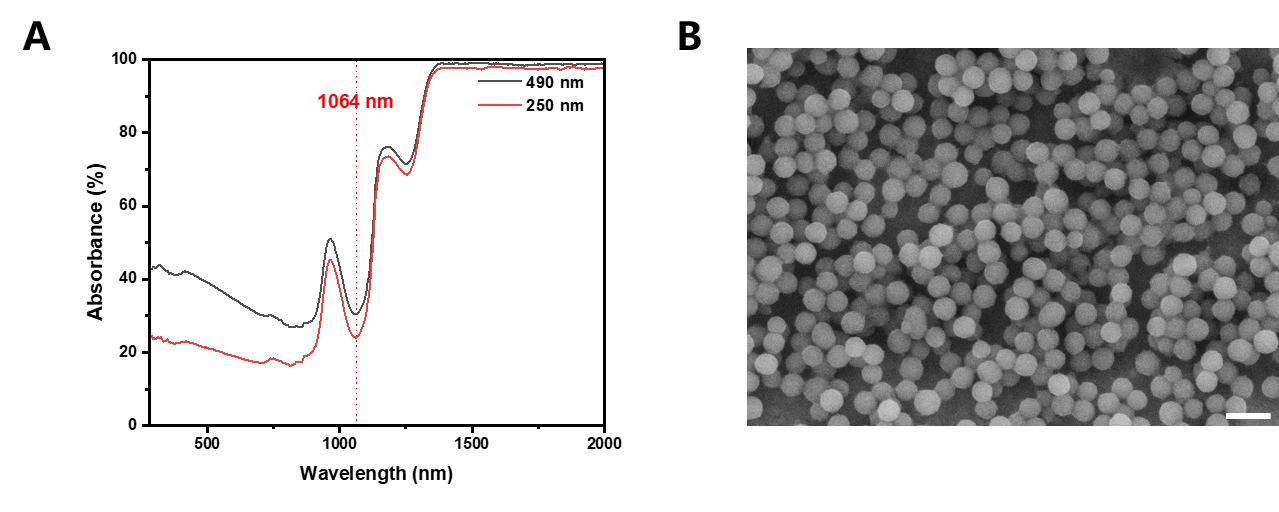


**Figure S9. (A)**Full-wavelength absorbance spectra of PDA NPs of different particle sizes, **(B)** SEM image of 250 nm PDA NPs. Scale bar, 500 nm


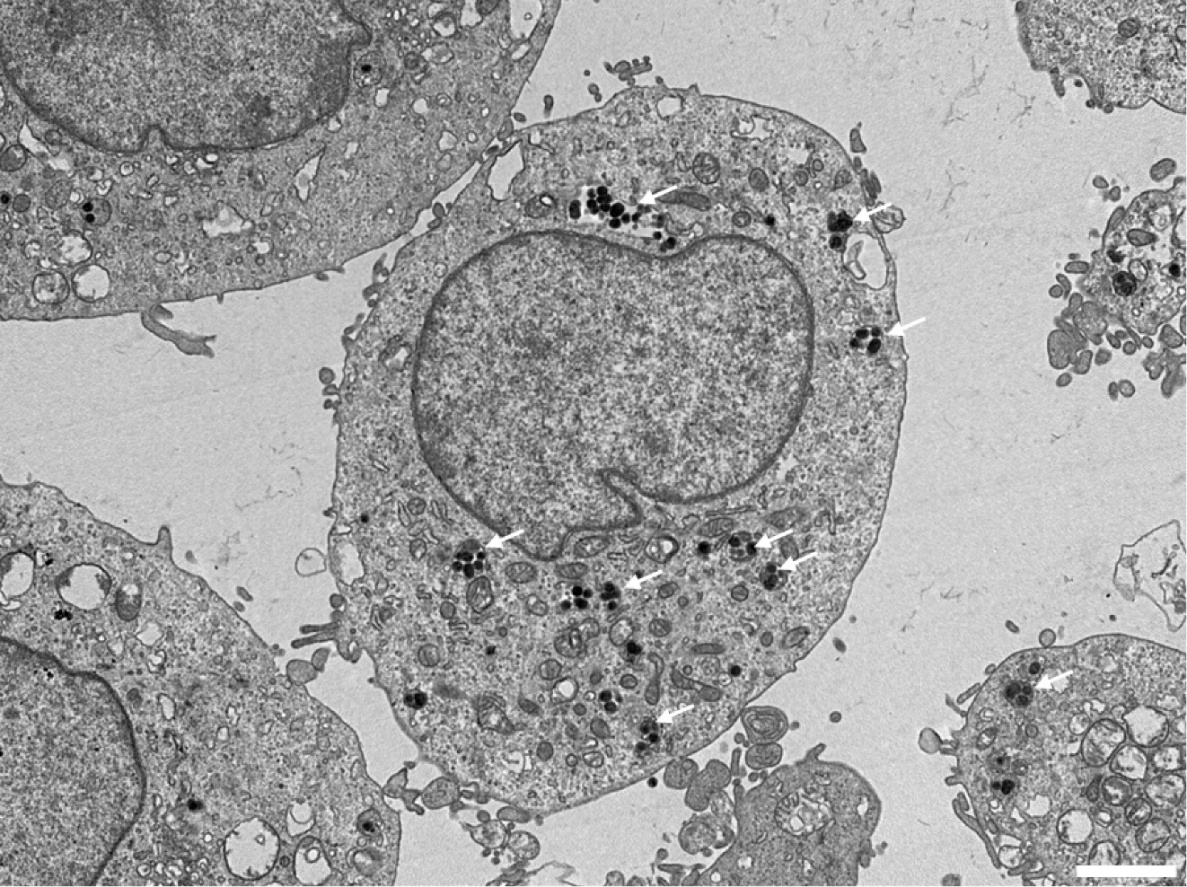


**Figure S10.** Transmission electron microscopy of PDA NPs with 150 nm size after 24h of co-incubation with PC12 cells. Scale bar, 2 μm.


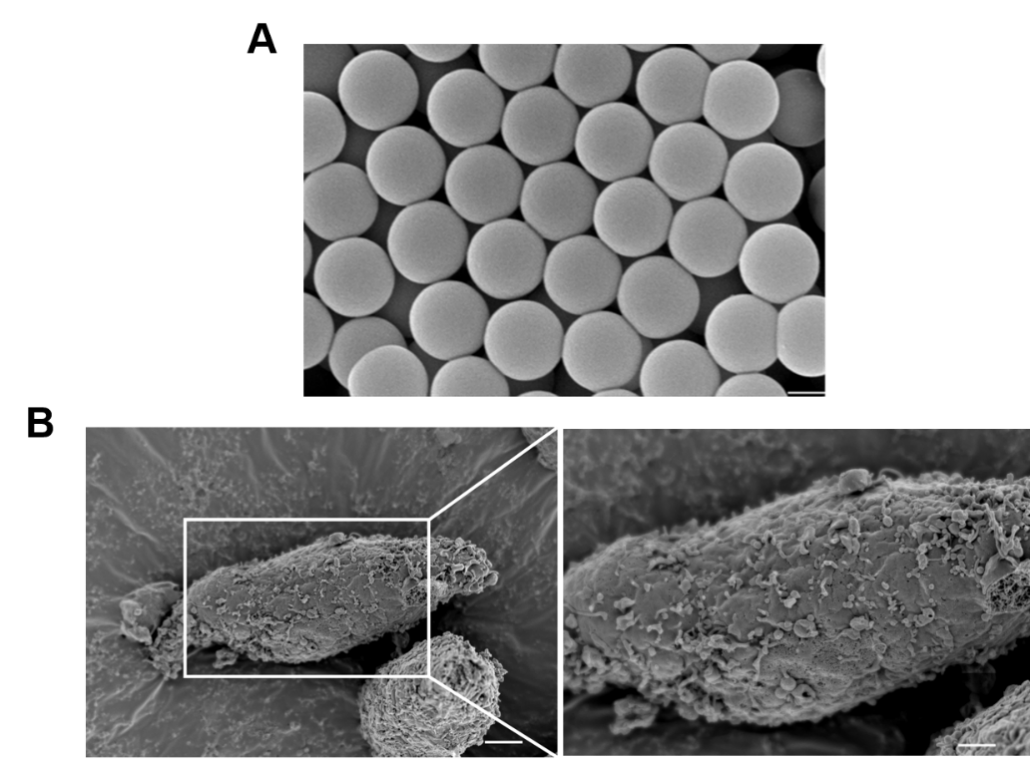


**Figure S11.** **(A)** Scanning electron microscope images of SiO_2_ NPs. Scale bar,200 nm. **(B)** Scanning electron micrograph of SiO_2_ NPs after 24h of co-incubation with PC12 cells. Scale bar,1 μm.

**Figure S12. The release amount of dopamine monomers by PDA NPs under NIR irradiation.** The mount of dopamine monomers by PDA NPs under different power NIR irradiation. (F_(2, 6)_=20.8, p=0.002), values are shown as mean ± SEM (n=3/group). Results were analyzed by one-way ANOVA with Dunnett’s post-hoc test. Statistically signiﬁcant diﬀerences are indicated by asterisk: ** *p* < 0.01.


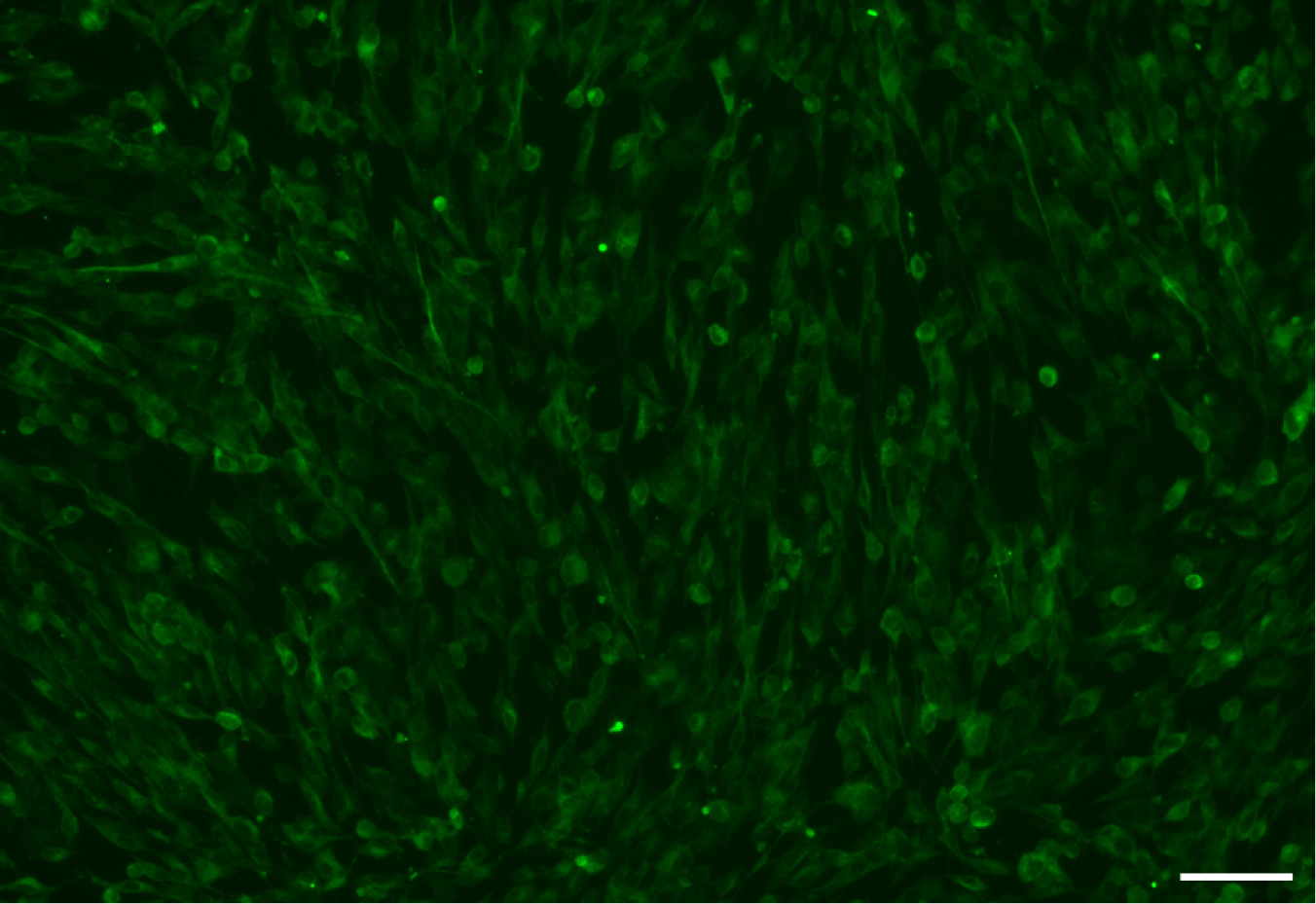


**Figure S13.** Representative immunofluorescence of TRPV1 with PC12 cells. Scale bar, 100 μm.


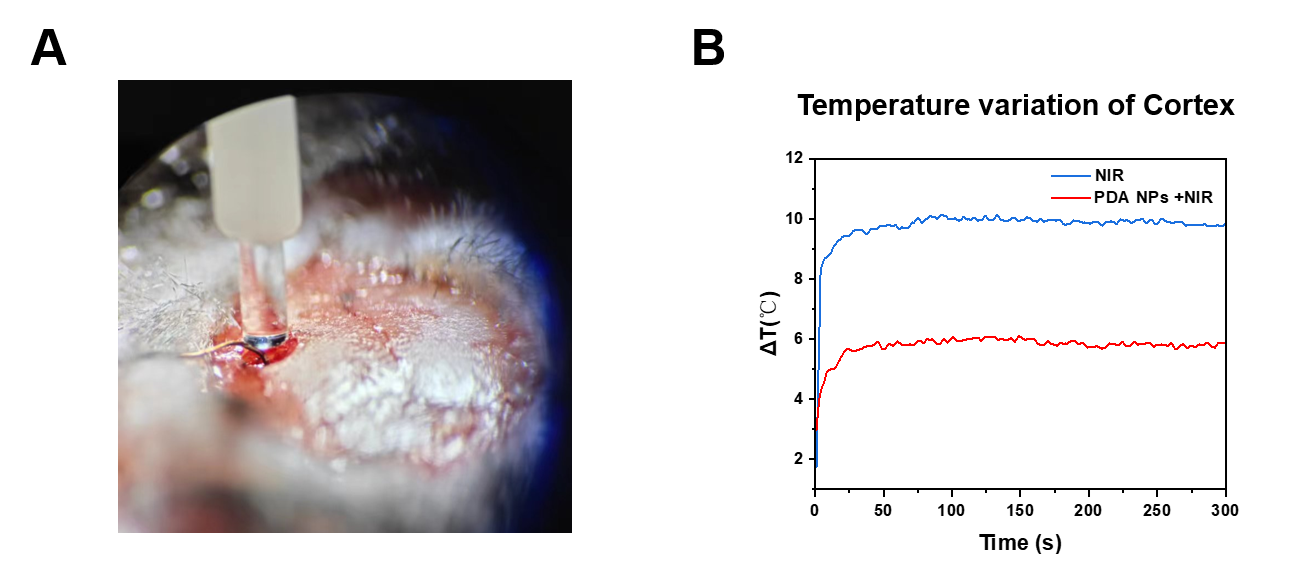


**Figure S14.** **(A)** Pictures of mice cortical temperature monitoring. **(B)** Temperature change curves in the cortical temperature of mice with or without PDA NPs into the DG region at 1064 nm laser irradiation for 300 seconds.


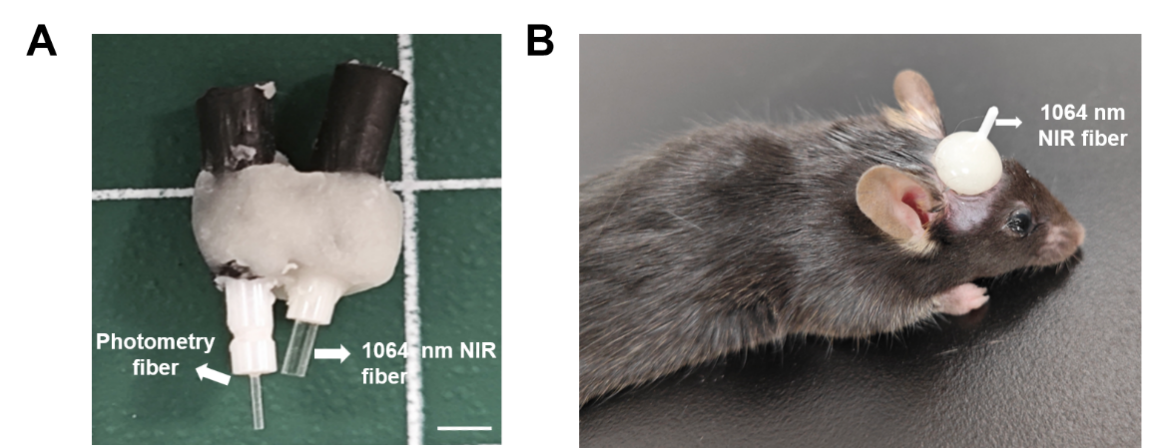


**Figure S15. (A)** Schematic of the fiber photometry setup for monitoring NIR-regulated calcium signal activity in the hippocampus. Scale bar, 2 mm. **(B)** Schematic of the therapeutic device for neuromodulation.


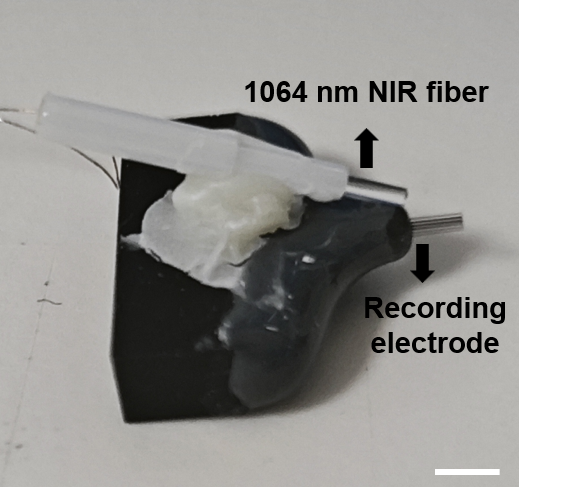


**Figure S16.** **Schematic of the electrode recording setup for monitoring NIR-regulated calcium signal activity in the hippocampu**s. Scale bar, 2 mm.


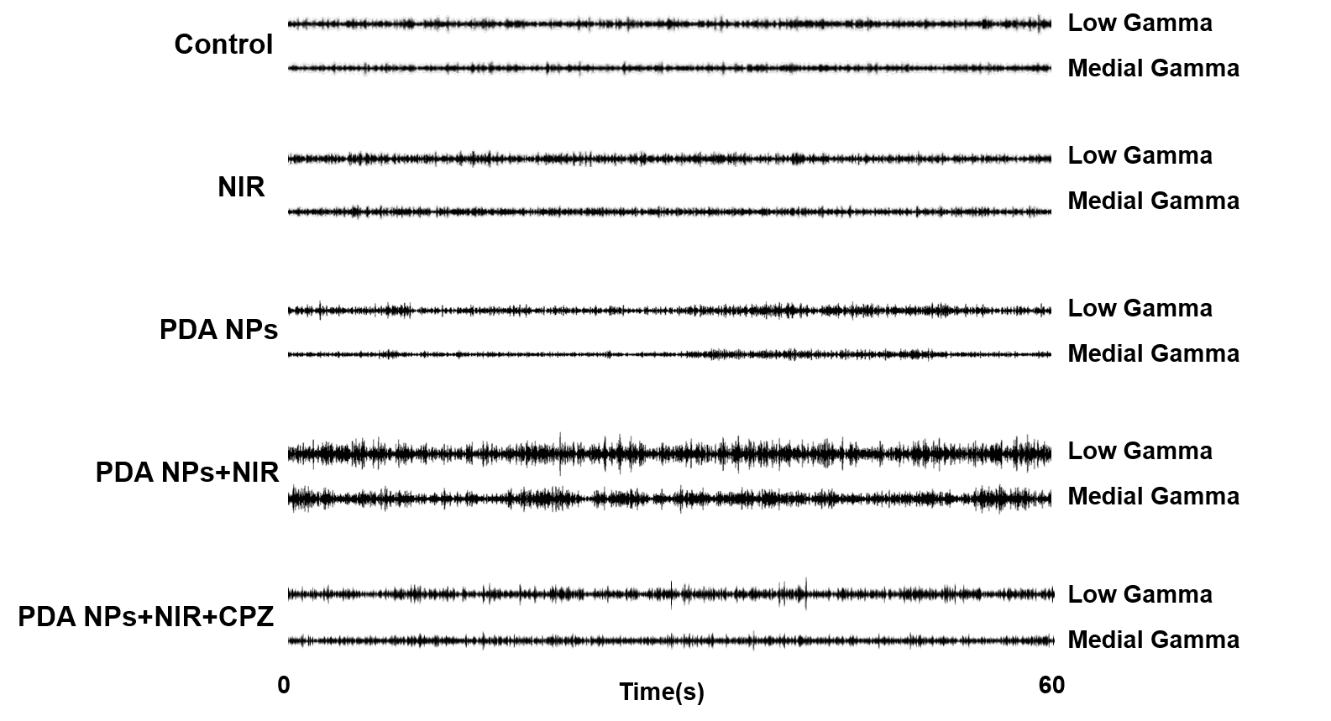


**Figure S17.** Representative power spectrum of filtered low gamma and medial gamma band of hippocampal DG region in control, NIR, PDA NPs, PDA NPs + NIR, PDA NPs + NIR + CPZ groups in normal mice.


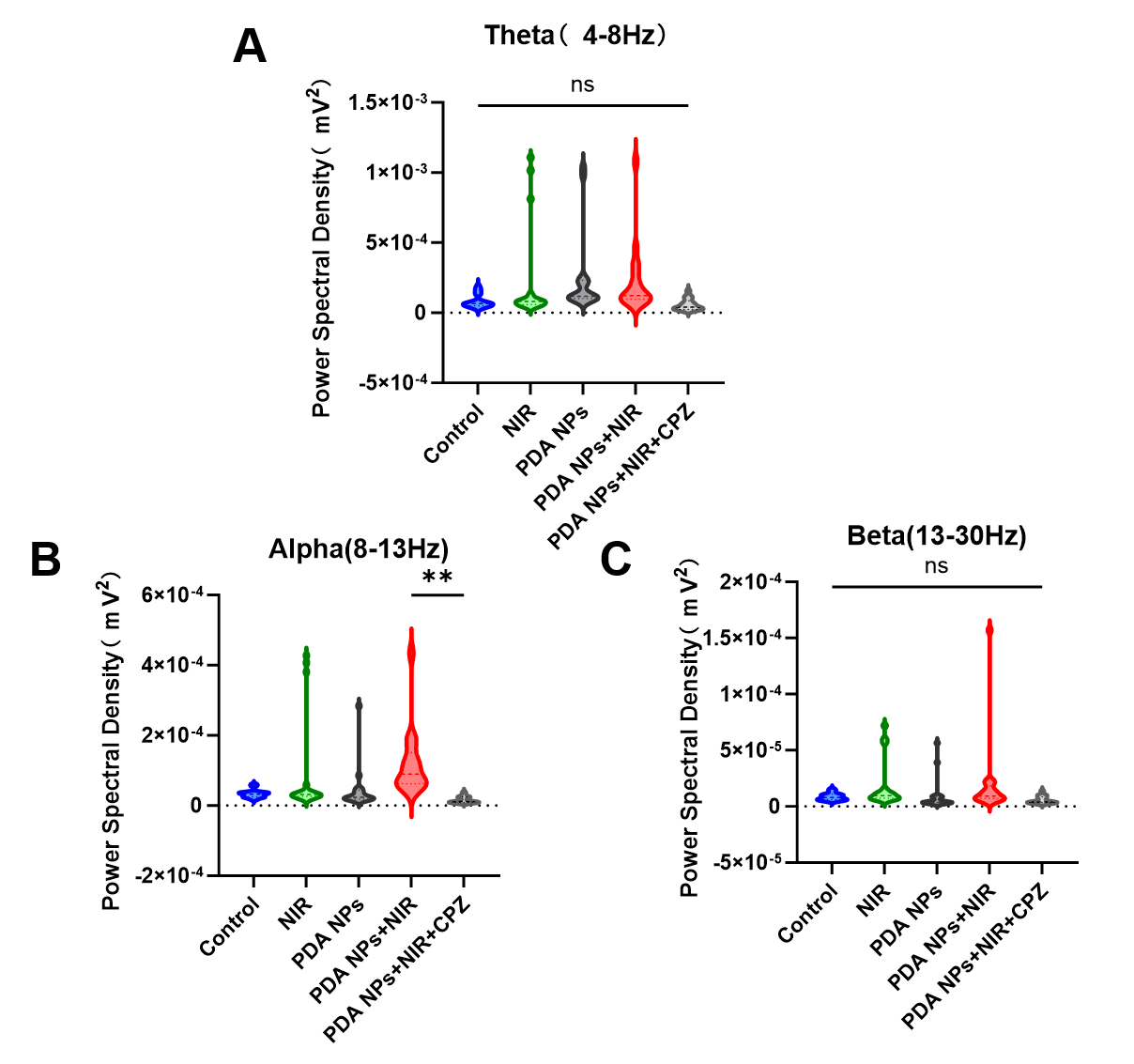


**Figure S18.** **(A)** Quantification of average theta (F_(4, 75)_= 2.405,p=0.0570)**, (B)**alpha(F_(4, 75)_= 4.429,p=0.0029)**,** and **(C)**beta(F_(4, 75)_=1.610,p=0.1806) oscillation in the DG in different groups. values are shown as mean ± SEM (n=16 from 4 mice/group). A, B, C) Results were analyzed by one-way ANOVA with Tukey's post-hoc test. Statistically signiﬁcant diﬀerences are indicated by asterisk: ***p* < 0.01.


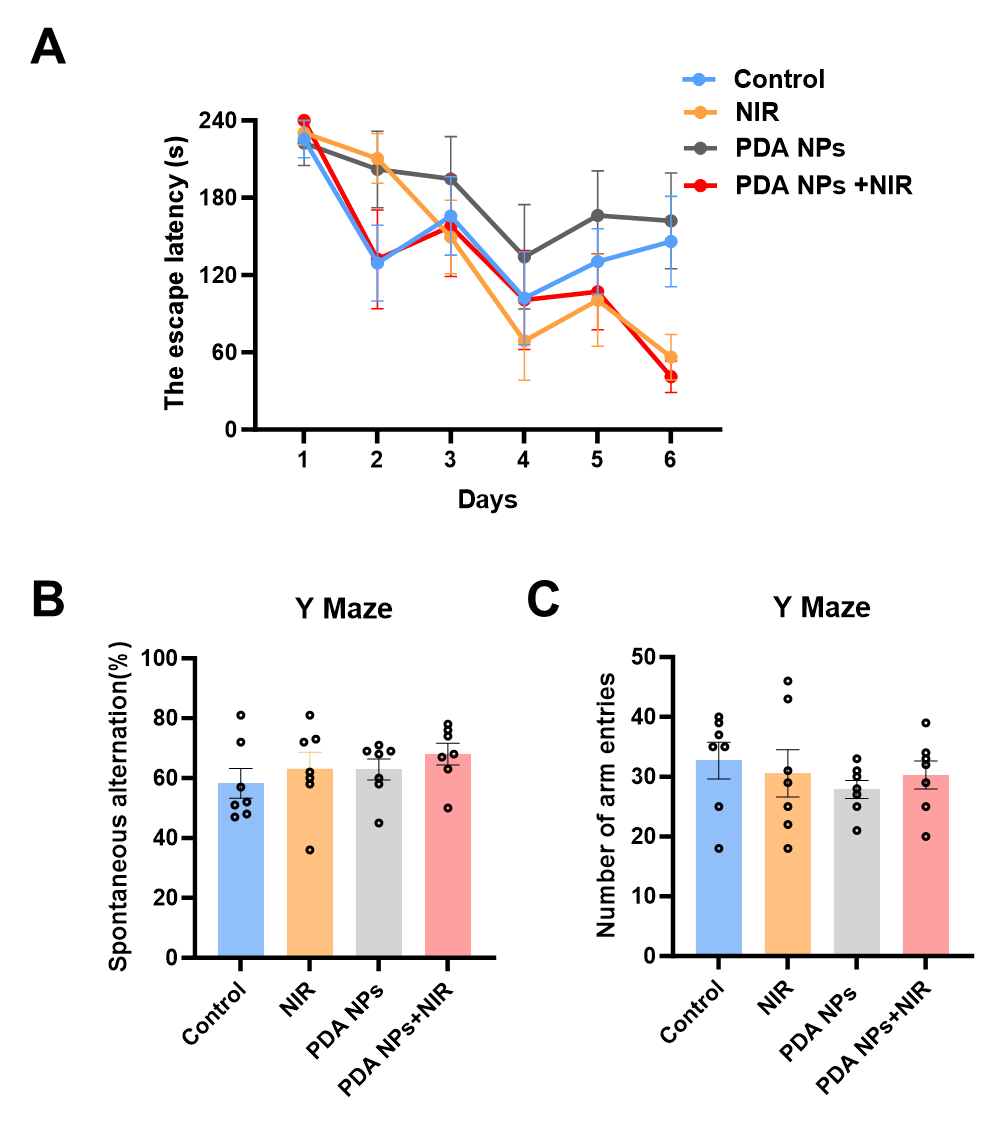


**Figure S19**. **(A)** Identify latency of the training and test period of mice during the Barnes maze test, values are shown as mean ± SEM (n=7 mice/group). **(B)** The spontaneous alternation in each group (F _(3, 24)_ = 0.7853, p=0.5139), values are shown as mean ± SEM (n=7 mice/group). **(C)** The number of arm entries in each group (F _(3, 24)_ = 0.4786, p=0.7002)**,** values are shown as mean ± SEM (n=7 mice/group). B, C) Results were analyzed by one-way ANOVA with Dunnett’s post-hoc test.


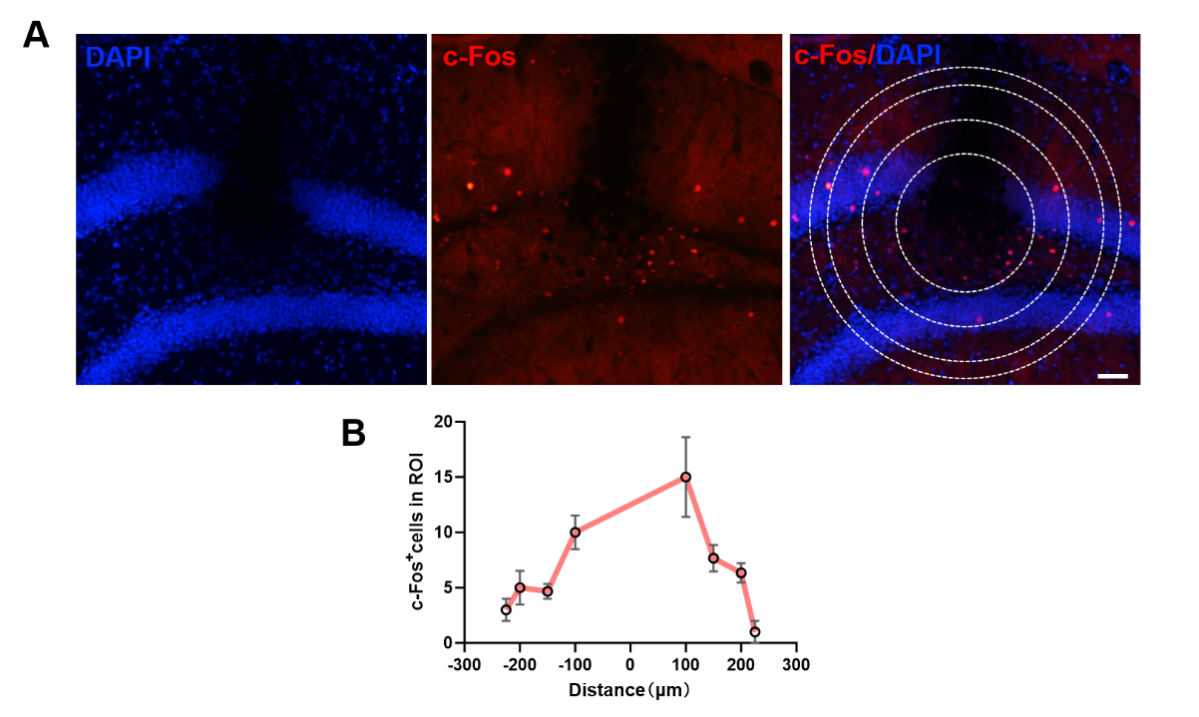


**Figure S20. The in vivo spatial resolution of the NIR neuromodulation strategy mediated by PDA NP. (A)** Representative immunofluorescence of c-Fos under PDA NP mediated NIR conditions in DG region. Scale bar, 50 μm. **(B)** The number of c-Fos positive cells under PDA NP mediated NIR conditions in DG region (n=3 mice/group).


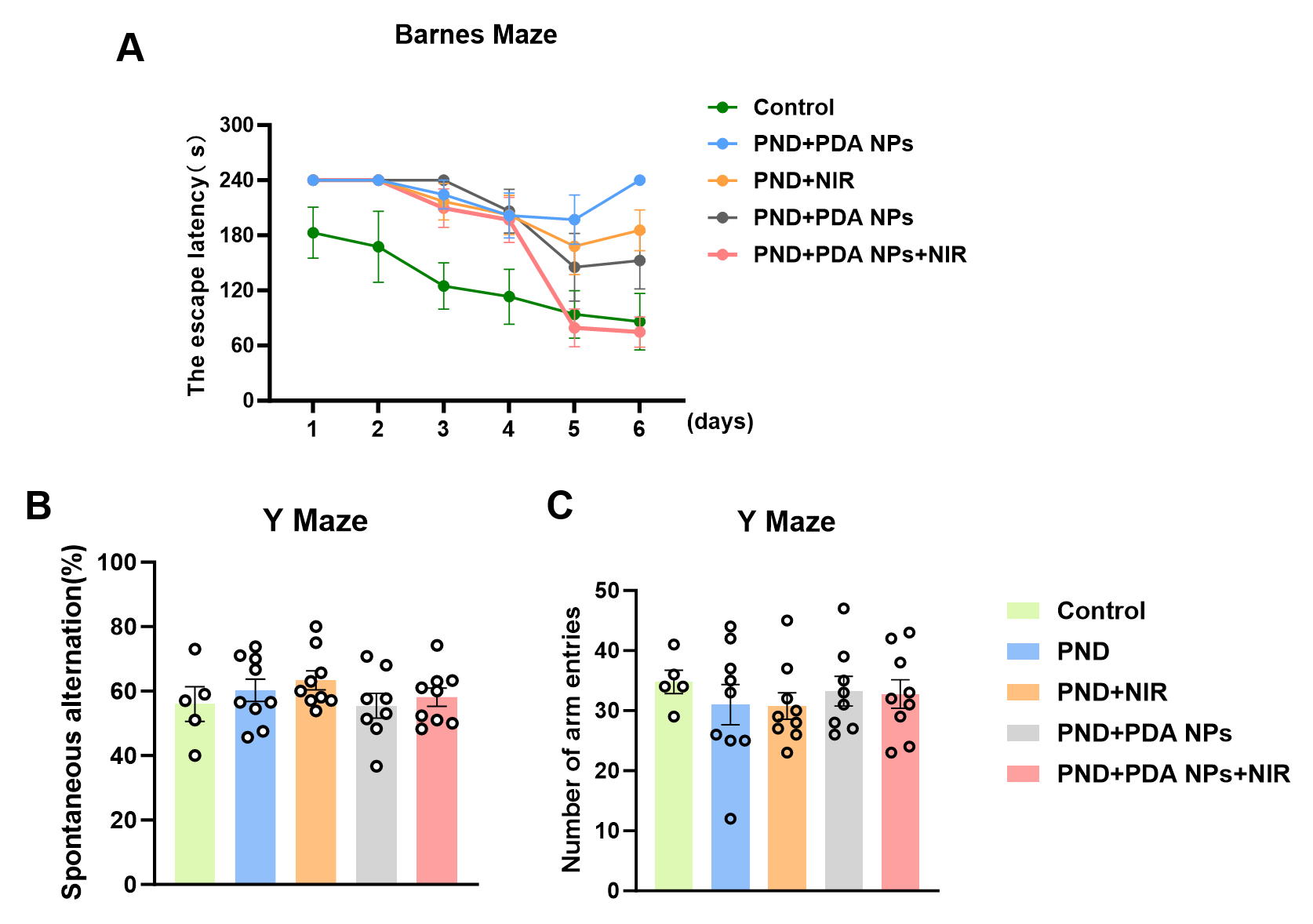


**Figure S21**. **(A)** Identify latency of the training and test period of mice during the Barnes maze test. Values are shown as mean ± SEM (n = 7 mice/group). **(B)** The spontaneous alternation in each group (F_(4, 35)_=0.8439, *p*=0.5069), values are shown as mean ± SEM (n = 5-9 mice/group). **(C)** The number of arm entries in each group (F_(4, 35)_=0.3371, *p*=0.8511) values are shown as mean ± SEM (n=5-9mice/group). B, C) Results were analyzed by one-way ANOVA with Dunnett’s post-hoc test.


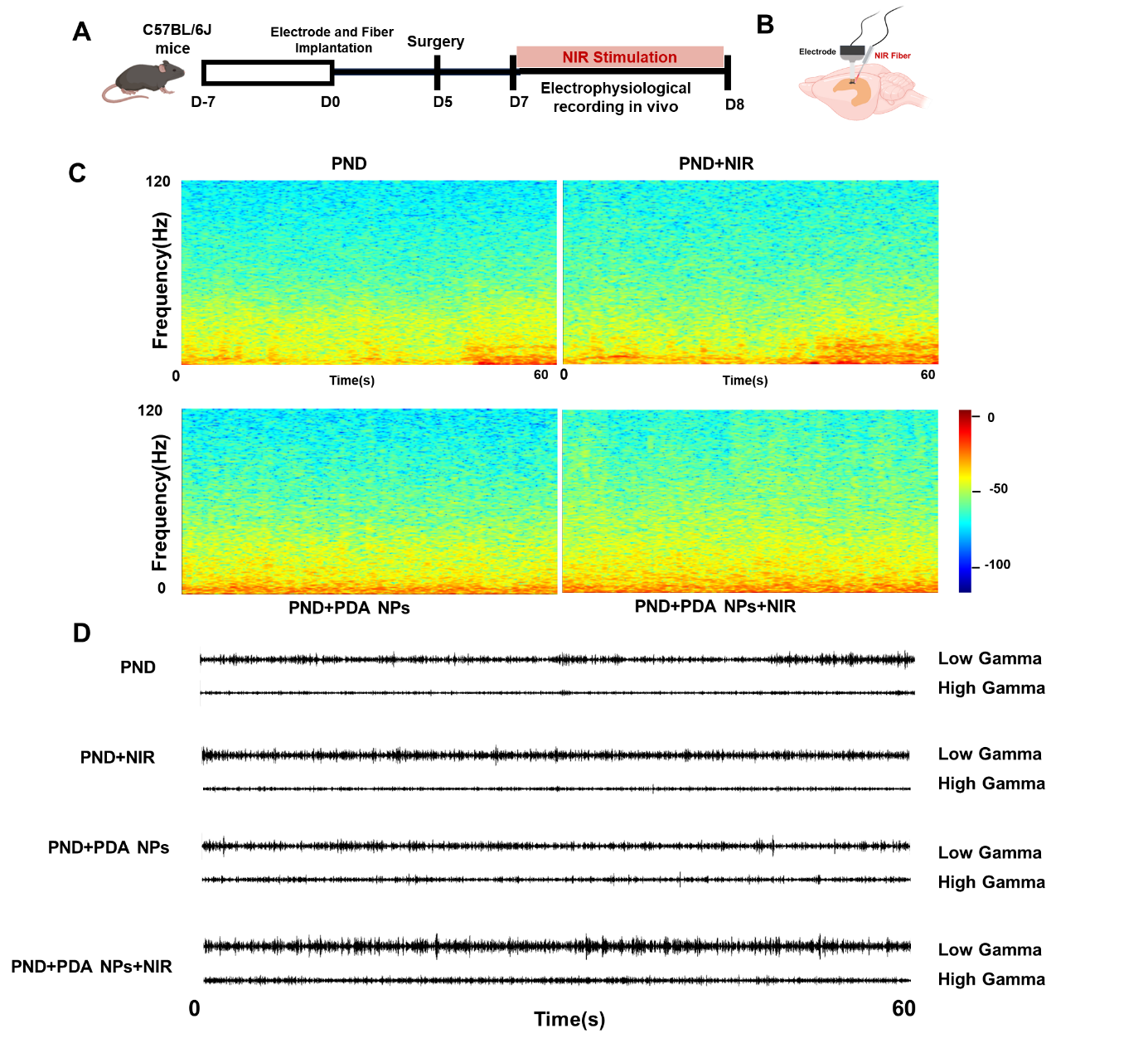


**Figure S22.** **PDA NP-mediated NIR enhanced gamma oscillations in the DG in PND mice. (A)** Schematic diagram of PND mice making in vivo electrophysiologic recordings. **(B)** Schematic diagram of electrode and fibre optic implantation. **(C)** Representative spectrogram analysis of local field potential in DG. **(D)** Representative power spectrum of filtered low gamma and medial gamma band of DG region of each group in PND mice.


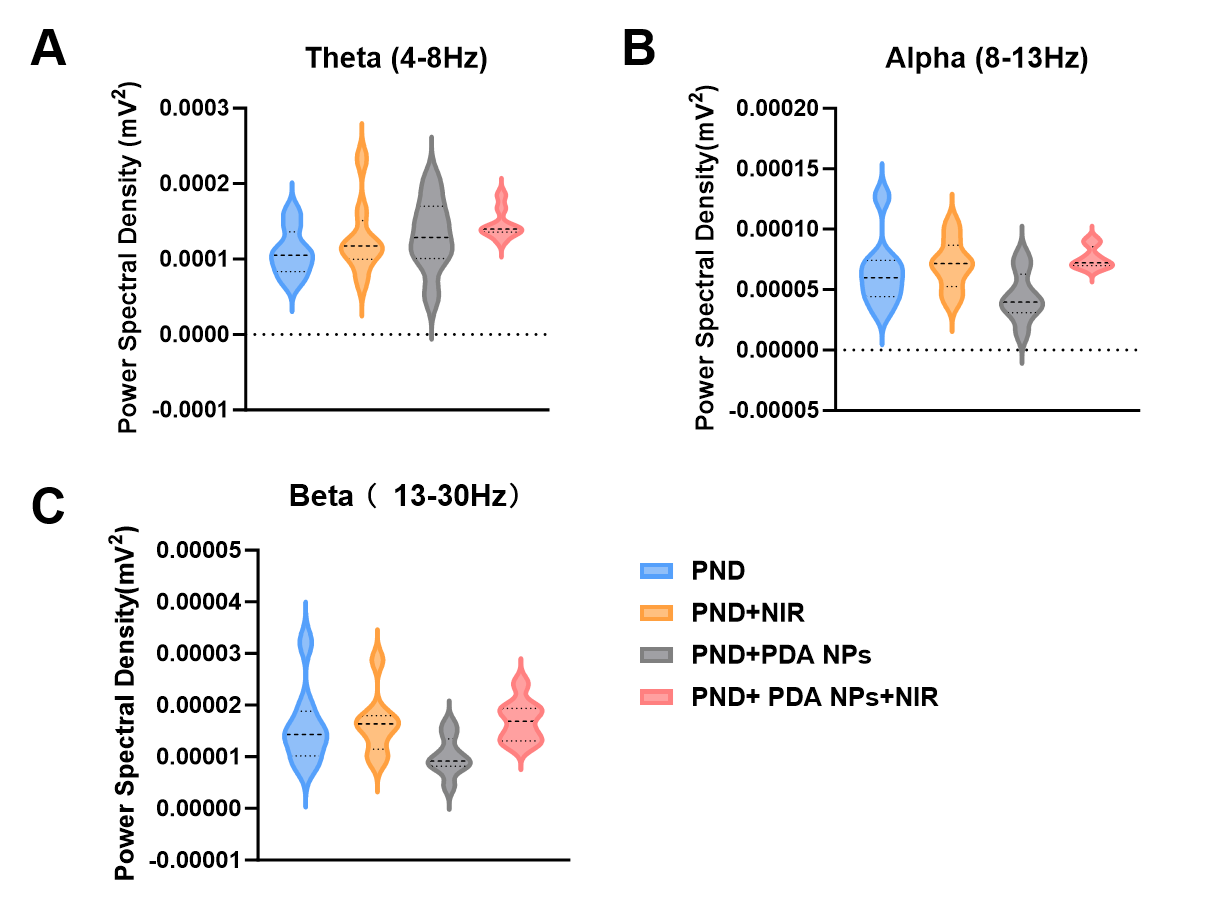


**Figure S23.** Quantification of average **(A)** theta (F_(3, 28)_= 1.237, *p*=0.3149) **(B)** alpha (F_(3, 28)_=3.533, *p*=0.0275) and **(C)** beta (F_(3, 28)_=2.622, *p*=0.0702) oscillation in the DG in different groups. A, B, C) Results were analyzed by one-way ANOVA with Dunnett’s post-hoc test, values are shown as mean ± SEM (n=8 from 4 mice/group).

**Table S1**

**Table S1.** **Optical properties of mouse head tissue type and PDA NPs at 1064 nm.** $\text{μ}_{\text{s}}$, scattering coefficient (1/mm);$\text{g}$, anisotropy factor; $\text{μ}_{\text{a}}$, absorption coefficient (1/mm); *n*, refractive index.^[1]^

|  | $\text{μ}_{\text{s}}$ | $\text{μ}_{\text{a}}$ | $\text{g}$ | *n* |
| --- | --- | --- | --- | --- |
| WM | 7.8 | 0.01 | 0.91 | 1.4 |
| GM | 7.8 | 0.01 | 0.91 | 1.4 |
| CSF | 0.01 | 0.0582 | 0.90 | 1.4 |
| Skull | 19.8 | 0.05 | 0.95 | 1.4 |
| Skin | 10 | 0.037 | 0.90 | 1.4 |
| PDA NPs | 7.8 | 0.25 | 0.91 | 1.4 |

**Table S2**

**Table S2.** **Bioheat Transfer Properties.** $C_{p}$(J/kg/K), Specific heat; $\rho$(kg/m^3^), density; $k$(W/m/K), tmermal conductivity; $Q_{m}$(W/m^3^), metabolic heat; $\omega_{b}$(1/s), blood perfusion; $\rho_{b}$ (kg/m^3^), blood density, $C_{b}$(J/kg/K), blood specific heat capacity.^[2,3]^

|  | $C_{p}$ | $\rho$ | $k$ | $Q_{m}$ | $\omega_{b}$ | $C_{b}$ | $\rho_{b}$ |
| --- | --- | --- | --- | --- | --- | --- | --- |
| Brain tissue | 3680 | 1035.5 | 0.52 | 15575 | 0.17467 | - | - |
| Blood | - | - | - | - | - | 3600 | 1057 |
| CSF | 4096 | 1007 | 0.57 | 0 | 0 | - | - |
| Skull | 1313 | 1908 | 0.32 | 70 | 0.000143 | - | - |
| Skin | 3391 | 1109 | 0.37 | 363 | 0.00143 | - |  |

**Table S3**

**Table S3. Comprehensive comparison of PDA NP-mediated NIR photothermal neuromodulation with magnetothermal neuromodulation strategies**

| **Neuro-modulation method** | **NPs**  **Synthesis Complexity** | **Virus required** | **Excitation**  **Source** | **Invasiveness** | **Spatial precision** | **Wiring** |
| --- | --- | --- | --- | --- | --- | --- |
| Magnetothermal  Stimulation^[4]^ | High | None | AMF | Minimally invasive | Moderate | Wireless |
| Magnetothermal  stimulation^[5]^ | Moderate | Required | AMF | Moderately invasive | High | Wireless |
| Magnetothermal genetic stimulation^[6]^ | Moderate-high | Required | AMF | Moderately invasive | High | Wireless |
| Chemomagnetic  Neuromodulation  ^[7]^ | High | None | AMF | Minimally invasive | Moderate | Wireless |
| Magnetothermal stimulation^[8]^ | Low | Required | AMF | Moderately invasive | Moderate | Wireless |
| Magnetothermal multiplexing^[9]^ | Moderate-high | Required | AMF | Moderately invasive | High | Wireless |
| PDA NP  -mediated NIR  photothermal  (This Work) | Very low | None | Near‑  infrared  light | Minimally invasive | High | Wire |

Note: NPs: Nanoparticles, AMF: Alternating magnetic field

**Reference:**

1. S. Yang, B. Zhang, M. Li, Z. Liu, X. Yang, Y. Li, T. Li, "Bridging animal and human: a 3D computational framework of light propagation in head models for transcranial photobiomodulation in Alzheimer's disease", Brain‐X 4 (2026): e70048.
https://doi.org/https://doi.org/10.1002/brx2.70048

2. Y. Shin, M. Yoo, H.-S. Kim, S.-K. Nam, H.-I. Kim, S.-K. Lee, S. Kim, H.-S. Kwon, Characterization of fiber-optic light delivery and light-induced temperature changes in a rodent brain for precise optogenetic neuromodulation. In Biomed. Opt. Express, 2016; Vol. 7, pp 4450.

3. A. R. Guillen, D. Q. Truong, P. C. Faria, B. Pryor, L. de Taboada, A. Datta, "High-Resolution Computational Modeling of Transcranial Photobiomodulation: Light Propagation and Thermal Effects", Neuromodulation: Technology at the Neural Interface 29 (2026):267.
https://doi.org/https://doi.org/10.1016/j.neurom.2025.06.003

4. G. Li, X. Qiao, Y. Zhao, D. Li, G. Zhang, X. Liu, F. Chen, H. Wang, H. Lu, J. Zhou, C. Wang, H. Fan, "Ferrimagnetic Vortex Nanorings Facilitate Efficient and Safe Deep‐Brain Magnetothermal Stimulation in Freely Moving Mice", Exploration 5 (2025): 20240118. <https://doi.org/10.1002/exp.20240118>

5. S. A. Hescham, P. H. Chiang, D. Gregurec, J. Moon, M. G. Christiansen, A. Jahanshahi, H. Liu, D. Rosenfeld, A. Pralle, P. Anikeeva, Y. Temel, "Magnetothermal nanoparticle technology alleviates parkinsonian-like symptoms in mice", Nat Commun 12 (2021):5569.
https://doi.org/10.1038/s41467-021-25837-4

6. R. Munshi, S. M. Qadri, Q. Zhang, I. Castellanos Rubio, P. Del Pino, A. Pralle, "Magnetothermal genetic deep brain stimulation of motor behaviors in awake, freely moving mice", Elife 6 (2017): e27069. <https://doi.org/10.7554/eLife.27069>

7. R. T. Guntnur, N. Muzzio, A. Gomez, S. Macias, A. Galindo, A. Ponce, G. Romero, "On-Demand Chemomagnetic Modulation of Striatal Neurons Facilitated by Hybrid Magnetic Nanoparticles", *Adv Funct Mater* 32 (2022**)**: 2204732. <https://doi.org/10.1002/adfm.202204732>

8. T. A. Le, M. P. Bui, J. Yoon, "Theoretical Analysis for Wireless Magnetothermal Deep Brain Stimulation Using Commercial Nanoparticles", Int J Mol Sci 20 (2019): 2873. <https://doi.org/10.3390/ijms20122873>

9. J. Moon, M. G. Christiansen, S. Rao, C. Marcus, D. C. Bono, D. Rosenfeld, D. Gregurec, G. Varnavides, P. H. Chiang, S. Park, P. Anikeeva, "Magnetothermal Multiplexing for Selective Remote Control of Cell Signaling", Adv Funct Mater 30 (2020): 2000577. https://doi.org/10.1002/adfm.202000577
